# Supplementary material for: Are trajectories of depressive symptoms during the first half of drug-sensitive pulmonary tuberculosis treatment associated with loss to follow-up? A secondary analysis of longitudinal data
Source: BMJ Open. 2023 Jul 19;13(7):e068235. doi: 10.1136/bmjopen-2022-068235 (PMC10357812; doi:10.1136/bmjopen-2022-068235)
Supplement: Supplementary data [file bmjopen-2022-068235supp001.pdf]

Supplemental Material 1: Code for performing the trajectory analysis using Mplus v8.0.

```
TITLE:
LCGA cesd0 to cesd3  Quadratic with 3 Classes

DATA: File is
"C:\Users\paulo\Google Drive\Doctorado\Second Try\cesd0-3\cesd3.dta.dat";
    LISTWISE=ON;

VARIABLE:
Names are
     id cesd0 cesd1 cesd2 cesd3 ;
     usevar = cesd0 cesd1 cesd2 cesd3 ;
     IDVARIABLE=id;
     CLASSES=C(3);
  Missing are all (-9999) ;

SAVEDATA: FILE is
"C:\Users\paulo\Google Drive\Doctorado\Second Try\cesd0-3\S2LCGA_0a3_L2.csv";
SAVE=CPROB;

ANALYSIS:
    TYPE=MIXTURE;
    STARTS = 500 10;
    STITERATIONS = 10 ;
    LRTBOOTSTRAP=50;

MODEL: %OVERALL%
I S Q| cesd0@0 cesd1@1 cesd2@2 cesd3@3;
I-S@0;

OUTPUT: SAMPSTAT  STANDARDIZED MOD (5.00) TECH4 TECH7 TECH11 TECH13 TECH14;
PLOT: SERIES = cesd0-cesd3(S); TYPE=PLOT3;
```


Ruiz-Grosso P, *et al. BMJ Open* 2023; 13:e068235. doi: 10.1136/bmjopen-2022-068235

Supplemental Material 2: Goodness of fit of the trajectories of depressive symptoms during the first half of fully sensitive TB treatment.

	2 trajectories		3 trajectories		4 trajectories	
	**Linear**	**Quadratic**	**Linear**	**Quadratic**	**Linear**	**Quadratic**
**AIC**	5448.7	5440	5400.3	5362.7	5351.9	5304.6
**BIC**	5480.9	5482.9	5443.2	5420	5405.6	5376.2
**SSA-BIC**	5452.3	5444.9	5443.2	5369.2	5358.1	5312.7
**Entropy**	0.803	0.828	0.864	0.858	0.909	0.913
**VLR Lrtest***	0.024	0.188	0.003	0.035	0.001	0.001
**BLRT****	<0.001	<0.001	<0.001	<0.001	<0.001	<0.001

*** Vuong–Lo–Mendell–Rubin likelihood ratio test for the null hypothesis that the model with the current number of trajectories is equal that the one with one less trajectory.**

**** Parametric Likelihood Ratio Test for the null hypothesis that the model with the current number of trajectories is equal that the one with one less trajectory using bootstrap.**

Ruiz-Grosso P, *et al. BMJ Open* 2023; 13:e068235. doi: 10.1136/bmjopen-2022-068235
