# Supplementary figures and images for: Are trajectories of depressive symptoms during the first half of drug-sensitive pulmonary tuberculosis treatment associated with loss to follow-up? A secondary analysis of longitudinal data
Source: BMJ Open. 2023 Jul 19;13(7):e068235. doi: 10.1136/bmjopen-2022-068235 (PMC10357812; doi:10.1136/bmjopen-2022-068235)

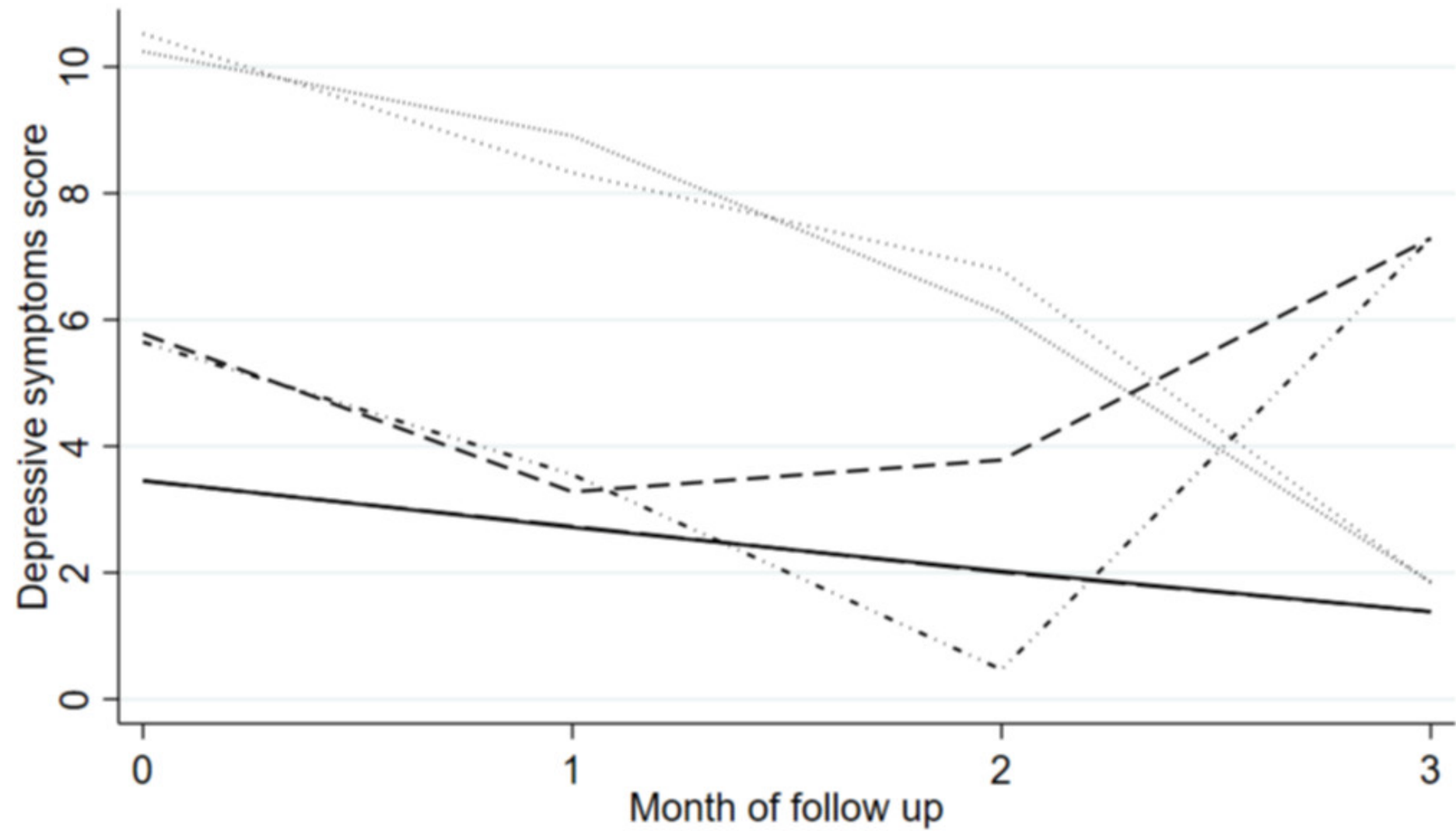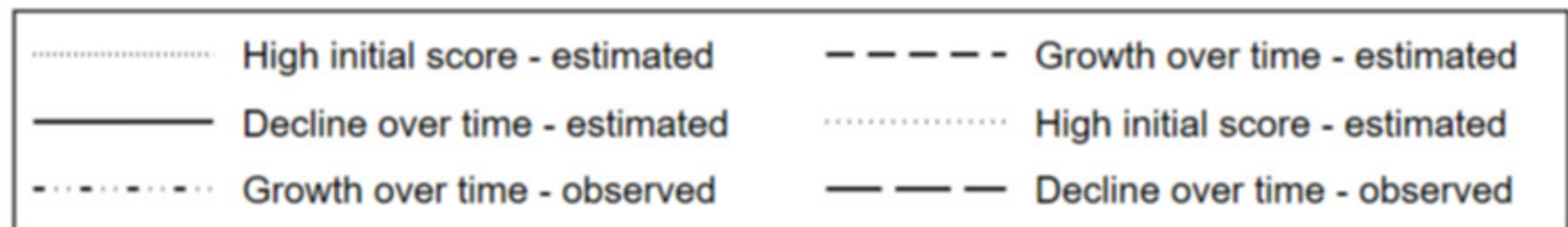

Supplement: Supplementary data [file bmjopen-2022-068235supp002.pdf]
