# Supplementary material for: Are trajectories of depressive symptoms during the first half of drug-sensitive pulmonary tuberculosis treatment associated with loss to follow-up? A secondary analysis of longitudinal data
Source: BMJ Open. 2023 Jul 19;13(7):e068235. doi: 10.1136/bmjopen-2022-068235 (PMC10357812; doi:10.1136/bmjopen-2022-068235)
Supplement: Author's manuscript [file bmjopen-2022-068235.draft_revisions.pdf]

BMJ Open is committed to open peer review. As part of this commitment we make the peer review history of every article we publish publicly available.

When an article is published we post the peer reviewers' comments and the authors' responses online. We also post the versions of the paper that were used during peer review. These are the versions that the peer review comments apply to.

The versions of the paper that follow are the versions that were submitted during the peer review process. They are not the versions of record or the final published versions. They should not be cited or distributed as the published version of this manuscript.

BMJ Open is an open access journal and the full, final, typeset and author-corrected version of record of the manuscript is available on our site with no access controls, subscription charges or pay-per-view fees (http://bmjopen.bmj.com).

If you have any questions on BMJ Open's open peer review process please email info.bmjopen@bmj.com

BMJ Open

Trajectories of depressive symptoms during the first half of drug sensitive tuberculosis treatment and loss to follow up in Lima, Peru.

Journal:	*BMJ Open*
Manuscript ID	bmjopen-2022-068235
Article Type:	Original research
Date Submitted by the Author:	07-Oct-2022
Complete List of Authors:	Ruiz-Grosso, Paulo; Universidad Peruana Cayetano Heredia, Medical School; Estado Peruano Ministerio de Salud, Programa de Control de Tuberculosis. Dirección de Redes Integradas de Salud Lima Norte.<br>Loret de Mola, Christian; Universidade Federal do Rio Grande do Sul, CPIS Grupo de Pesqueisa e Inovação em Saúde<br>Otero, Larissa ; Universidad Peruana Cayetano Heredia, Instituto de Medicina Tropical Alexander von Humboldt<br>Ugarte-Gil, Cesar; Universidad Peruana Cayetano Heredia, Facultad de Medicina Alberto Hurtado; Universidad Peruana Cayetano Heredia, Instituto de Medicina Tropical Alexander von Humboldt
Keywords:	Tuberculosis < INFECTIOUS DISEASES, Depression & mood disorders < PSYCHIATRY, EPIDEMIOLOGY

SCHOLARONE™
Manuscripts

I, the Submitting Author has the right to grant and does grant on behalf of all authors of the Work (as defined in the below author licence), an exclusive licence and/or a non-exclusive licence for contributions from authors who are: i) UK Crown employees; ii) where BMJ has agreed a CC-BY licence shall apply, and/or iii) in accordance with the terms applicable for US Federal Government officers or employees acting as part of their official duties; on a worldwide, perpetual, irrevocable, royalty-free basis to BMJ Publishing Group Ltd ("BMJ") its licensees and where the relevant Journal is co-owned by BMJ to the co-owners of the Journal, to publish the Work in this journal and any other BMJ products and to exploit all rights, as set out in our licence.

The Submitting Author accepts and understands that any supply made under these terms is made by BMJ to the Submitting Author unless you are acting as an employee on behalf of your employer or a postgraduate student of an affiliated institution which is paying any applicable article publishing charge ("APC") for Open Access articles. Where the Submitting Author wishes to make the Work available on an Open Access basis (and intends to pay the relevant APC), the terms of reuse of such Open Access shall be governed by a Creative Commons licence – details of these licences and which Creative Commons licence will apply to this Work are set out in our licence referred to above.

Other than as permitted in any relevant BMJ Author's Self Archiving Policies, I confirm this Work has not been accepted for publication elsewhere, is not being considered for publication elsewhere and does not duplicate material already published. I confirm all authors consent to publication of this Work and authorise the granting of this licence.

For peer review only

Title:

Trajectories of depressive symptoms during the first half of drug sensitive tuberculosis treatment and loss to follow up in Lima, Peru.

Running title:

Depression trajectories and loss to follow up

Authors

Paulo Ruiz-Grosso 1, 2

Christian Loret de Mola 3

Larissa Otero 1, 2

Cesar Ugarte-Gil 1, 2

1 Instituto de Medicina Tropical Alexander von Humboldt, Universidad Peruana Cayetano Heredia, Lima, Peru

2 Facultad de Medicina, Universidad Peruana Cayetano Heredia, Lima, Peru

3 Grupo de pesquisa e inovação em saúde do programa de pós-graduação em saúde pública da universidade federal do Rio Grande, Brazil

Corresponding autor:

Paulo Ruiz-Grosso

Universidad Peruana Cayetano Heredia

Av. Honorio Delgado 430, San Martín de Porres

Email: paulo.ruiz@upch.pe

For peer review only - http://bmjopen.bmj.com/site/about/guidelines.xhtml

Text word count:

Total: 4565. Main body (including tables): 3039.

Number of references: 24.

Number of tables: 3.

Number of graphics: 2.

For peer review only

For peer review only - http://bmjopen.bmj.com/site/about/guidelines.xhtml

ABSTRACT

INTRODUCTION: Depression is associated with poorer outcomes during tuberculosis (TB) treatment. Depressive symptoms (DS) might present diverse trajectories during TB treatment, and some of them might be associated to a higher risk of loss to follow up (LTFU). We aim to identify trajectories of DS on the first half of treatment for fully sensitivity pulmonary TB (PTB) and their association with LTFU during its second half.

METHODS: Secondary analysis of longitudinal data. Measures of depressive symptoms were completed on diagnosis of PTB and monthly until its culmination, diagnosis of multi-drug resistant TB, LTFU or death. Trajectories of DS were identified using latent class growth analysis and its association with LTFU tested using logistic regression.

RESULTS: Three trajectories of DS, “Declining” “Growth” and “High” were presented in 68.7, 20 and 11.32% of individuals on the first half of PTB treatment, respectively. Compared to those with a “Declining”, those with “Growth” trajectory had higher chances of LTFU on the second half of PTB treatment controlling for sociodemographic and at least weekly alcohol use (OR=3.9; IC95%: 1.09-13.97).

CONCLUSION: A trajectory of increasing DS on the first half of PTB seems to be related to higher chances of LTFU during its second half.

STRENGTHS AND LIMITATIONS OF THIS STUDY

- We used monthly longitudinal measures of depressive symptoms starting from the time of diagnosis of full sensitivity TB.
- The trajectories (patterns of change) of depressive symptoms were extracted from the data using Latent Class Growth Analysis.
- Sample size and the relatively low incidence of the loss to follow up does not allow for precise confidence intervals.
- A locally validated, 5 item version of the CES-D scale was used to measure depression, however there is no information on the reliability of the scale when used multiple times in a relatively short period of time.

For peer review only - http://bmjopen.bmj.com/site/about/guidelines.xhtml

INTRODUCTION

Tuberculosis (TB) was the first cause of death due to infectious diseases worldwide, with 1.2 million deaths caused by TB in 2019, disproportionately affecting developing countries. After the onset of the COVID-19 pandemic, the detection of new cases of TB dropped, being the first period of increase in mortality due to TB since 2015, representing the second cause of death due to infectious diseases around the globe. The emergence of multidrug resistant strains (MDR) is an important challenge for the TB control, with 3% of cases detected around the globe starting treatment for MDR or XDR-TB. In Peru, 7.7% of cases starting treatment are MDR or XDR-TB and is considered one of the countries at a higher risk[1].

In Peru, treatment for full sensitive and MDR or XDR last for at least 6 and 24 months, respectively. Failure or poor adherence during treatment for full sensitivity TB increases the risk for developing MDR or XDR strains, making it more expensive, longer, and uncomfortable for the patient [2,3]. Failure and loss to follow up (LTFU) are considered important causes of non-successful TB treatment, accounting for 10 to 17 % of the cases of full sensitivity TB and 38% of MDR-TB [4,5]. It has been associated with lower level of education, receiving treatment for MDR-TB, and to comorbid depressive and substance use disorders [5–9].

Depressive disorders are more frequent in persons treated for TB than in general population, with a frequency of symptoms suggestive of major depressive episode (MDE) that ranged from 38% at the start of TB treatment and 14% at the end of it, while it was estimated in 7% in population-based studies in Lima[10]. In persons treated for MDR-TB, 58% and 33% had MDE at the start and end of the treatment, respectively [11]. Depressive symptoms corresponding to more severe forms such as MDE were associated to death (OR=2.85; IC95%: 1.52-5.36) and LTFU (OR=4.26; IC95%: 2.33-7.79) during treatment for TB, suggesting that they might be a target for interventions to improve TB treatment outcomes [12].

In this regard, the identification of subgroups of higher risk during the first half (FH) of treatment might allow more robust, specialized an expensive intervention in smaller groups. We analyzed data from a cohort of patients during drug sensitive TB treatment

For peer review only - http://bmjopen.bmj.com/site/about/guidelines.xhtml

to identify trajectories (patterns of change over time) of depressive symptoms associated to LTFU to identify high risk groups for these outcomes in which to explore opportunities for interventions.

For peer review only

For peer review only - http://bmjopen.bmj.com/site/about/guidelines.xhtml

METHODS

This is an analysis of data from a sub-sample of a cohort study designed to follow up patients on drug-sensitive pulmonary TB treatment in Lima, Peru. The original study took place at primary or secondary care public health centers and collected information on depressive symptoms monthly from TB diagnosis to the end of TB treatment 6 months after, death, LTFU or referral to another center. The aim of the study was to study risk factors for negative outcomes during TB treatment, such as death and default.

Individuals that participated in the study lived in San Juan de Lurigancho, a district on the East side of Lima, which is the most populated district in Peru, and are usually migrants or descendants of migrants of a large migration from the Andean regions of Peru [13]. The population ranges from middle class to poverty and have one of the larger burdens of TB in Peru[14]. Individuals younger than 18-year-old, those with a former diagnosis of TB, pregnant, illiterate, or with any mental of physical condition that would hinder the informed consent process were excluded from the study.

We included information of individuals that had at least four complete measurements of depressive symptoms (baseline and three-monthly follow ups) and excluded those diagnosed with MDR-TB during follow up. The main outcome variable was LTFU during the second half (SH) of TB treatment defined as the interruption of antituberculosis treatment for at least 30 days without medical indication. Our main predictor variable was trajectories (patterns of change over time) of depressive symptoms during the FH of TB treatment, identified using the score of 5 items, locally validated version of the CES-D in Spanish [15]. Participants completed the self-rating scale at the start of TB treatment and monthly afterwards.

We analyzed the data in two steps. First, we used latent class growth analysis (LCGA) to identify significant variations on the pattern of depressive symptoms score across the first 3 months of follow up [16]. We explored linear and quadratic models for trajectories, and used entropy, root mean squared error of approximation (RMSEA), comparative fix index (CFI), Akaike (AIC), Bayesian (BIC) information criteria, the *Vuong–Lo–Mendell–Rubin* and *Boostraped Likelihood Ratio Tests* to select the model which best fitted the

For peer review only - http://bmjopen.bmj.com/site/about/guidelines.xhtml

data. This step provided us with the information on the trajectories (slope, linear and quadratic terms) as well as assigning each participant with a trajectory.

On the second step, we used logistic regression to test the hypothesis that, individuals that presented worse trajectories of depressive symptoms during the FH of TB treatment were more likely to LTFU during SH than those with more favorable trajectories. Multiple logistic regression was used to adjust for potential confounders, including sex, age, marital, education, and employment status. At least weekly alcohol use at TB diagnosis (defined as drinking alcoholic beverages at least once per week) was analyzed to explore its role as a potential confounder by adjusting and as an effect modifier using stratification. Mplus 8.0 and Stata 15 were used for data analysis.

Research Ethics Approval

Ethical approval for this study was obtained from the Universidad Peruana Cayetano Heredia IRB, under review number (SIDISI number) 203884. All participants participated in the informed consent process and provided informed consent prior to enrollment in the study. For the purposes of this study, the data was received without any information that could identify the participants. Treating physicians were informed of the depression status of each participant.

Patient and Public Involvement

Neither patients nor the public were involved in the design, conduct, reporting or dissemination of this research

For peer review only

RESULTS

Of 344 participants, 34 were excluded because MDR-TB, and 34 because of missing data on the depression score. Of the 276 participants analyzed, 13 (4.9%) were lost to follow up during the SH of TB treatment. The mean age for participants was 29.6 (sd=12.9), 145 (54.7%) were male, and most were single (155, 58%). A total of 112 (42.3%) completed secondary education (high school), while 192 (72.5%) were unemployed when starting of TB treatment. Details of the sample might be found in Table 1.

Table 1. Sample characteristics of persons following fully sensitive TB treatment in Lima, Peru. (n=265)

		N (%)
**Male Sex**		145 (54.7)
**Age at enrollment***		29.6 (12.9)
**Marital status**		
	Single	155 (58.5)
	Married or cohabitant	90 (34.0)
	Divorced or widowed	20 (7.5)
**Attained education**		
	Incomplete elementary	20 (7.5)
	Complete elementary	75 (28.3)
	Complete secondary	112 (42.3)
	Superior education	58 (21.9)

For peer review only - http://bmjopen.bmj.com/site/about/guidelines.xhtml

**Currently employed**	73 (27.55)
**Trajectories for depressive symptoms during second half of treatment.**	
Decline over time	182 (68.7)
Growth over time	53 (20)
High initial score with decline	30 (11.32)
**Negative outcomes**	
Death	0 (0.0%)
Loss to follow up	13 (4.91)
Total	13 (4.91)

* mean; IC95%: 95% confidence interval

Identification of trajectories for depressive symptoms during the FH of TB treatment

Two quadratic solutions with 3 and 4 identified trajectories presented acceptable goodness of fit parameters (See Table 2). The first identified 3 trajectories: 1) “Decline over time of depressive symptoms” (69%), 2) “Growth over time” (20%) and 3) “High initial score with a decline over time” (11%). The second identified 4 trajectories: 1) “Decline over time of depressive symptoms” (65%), 2) “Slow growth over time” (21%), 3) “Rapid Growth over time” (5%) and 4) “High initial score with decline over time” (9%). Both these models were found significantly different from a solution with one less trajectory in both *Vuong–Lo–Mendell–Rubin* (p=0.035 and 0.001 for the 3 and 4 trajectories models, respectively) and the *Boostraped Likelihood Ratio Test* (p<0.001 for both). Details on the goodness of fit estimates and estimated parameters of solutions are found on Supplemental Material 1, while graphics of the trajectories might be found in Figures 1 and 2.

For peer review only - http://bmjopen.bmj.com/site/about/guidelines.xhtml

We selected the 3 trajectories solution for the next part of analysis because the 4 trajectories model showed two trajectories in which the proportion of participants were less than 10% of the sample, and being both growing trajectories very alike, a 3 trajectories models was more parsimonious.

Table 2. Parameters of estimated trajectories of depressive symptoms during the first half of fully sensitive TB treatment.

	2 trajectories		3 trajectories		4 trajectories	
	**Lineal**	**Quadratic**	**Lineal**	**Quadratic**	**Lineal**	**Quadratic**
Trajectory 1						
% of class	0.18	0.85	0.08	**0.12**	0.05	0.21
Intercept	9.40*	3.87	4.70*	**10.24***	3.92*	5.25*
Slope	´-1.74*	-1.56	1.24*	**-0.6**	2.15*	-3.53*
Quadratic term	NA	0.37	NA	**-0.7**	NA	1.224*
Trajectory 2						
% of class	0.82	0.15	0.79	**0.2**	0.22	0.09
Intercept	3.33	9.38	3.4	**5.8**	3.65*	10.67*
Slope	´-0.44*	-0.42	´-0.50	**´-4.00***	0.58*	-0.76
Quadratic term	NA	-0.56	NA	**1.50***	NA	-0.71
Trajectory 3						
% of class	NA	NA	0.13	**0.69**	0.09	0.05
Intercept	NA	NA	10.87*	**3.46***	11.97*	5.97*

Slope	NA	NA	´-2.77*	**´-0.77***	´-3.34*	´-2.82
Quadratic term	NA	NA	NA	**0**	NA	1.46*
Trajectory 4						
% of class	NA	NA	NA	NA	0.64	0.65
Intercept	NA	NA	NA	NA	3.57*	3.48
Slope	NA	NA	NA	NA	´-0.84*	´-0.67*
Quadratic term	NA	NA	NA	NA	NA	´-0.05*

* $p<0.05$

Association between trajectories of depressive symptoms on the FH and negative outcomes during the SH of TB treatment.

Compared to those with “Decline over time of depressive symptoms”, those with “Growth over time” during the FH of TB treatment had 3.74 (IC95%: 1.15-12.14) odds of LTFU during the SH. This remained significant when controlling for age, sex, at least weekly use of alcohol, marriage status, education, and job status (OR=3.90; IC95%: 1.09-13.97). No association was found for individuals with a high initial score with decline over time trajectory and LTFU (OR=1.28; IC95%: 0.13-12.54). Details of bivariate analyses can be found on Table 3.

Table 3: Variables associated to negative outcomes of TB during the second half of fully sensitive TB treatment.

Variables	Simple regression			Multiple regression		
	OR	IC95%	p	OR	IC95%	p
Trajectories of depressive symptoms during the first three months of fully sensitivity TB treatment.						

For peer review only - http://bmjopen.bmj.com/site/about/guidelines.xhtml

Decline over time	Ref.	-	-	Ref.	-	-
Growth over time	3.74	1.15 - 12.14	0.028	3.9	1.09 - 13.97	0.036
High initial score with decline	1.01	0.18 - 8.71	0.992	1.28	0.13 - 12.54	0.829
**Frequent alcohol use**	2.52	1.01 - 6.3	0.046	3.18	0.87 - 11.62	0.08
**Male sex**	1.35	0.54 - 3.22	0.498	2.21	0.50 - 9.75	0.296
**Age (years)**	1.01	0.97 - 1.04	0.742	0.98	0.92 - 1.05	0.653
Marital Status						
Single	Ref.	-	-	Ref.	-	-
Married or cohabitant	1.35	0.54 - 3.38	0.518	1.86	0.43 - 8.09	0.405
Divorced or widowed	1.92	0.50 - 7.40	0.343	2.35	0.33 - 16.70	0.392
**Attained education**						
Incomplete elementary	Ref.	-	-	Ref.	-	-
Complete elementary	0.78	0.15 - 4.11	0.766	0.97	0.07 - 13.05	0.983
Complete secondary	0.95	0.19 - 4.63	0.948	1.47	0.11 - 18.74	0.768
Superior education	0.92	0.17- 5.07	0.921	2.41	0.15 - 39.50	0.538
**Currently employed**	0.69	0.25 - 1.93	0.482	0.96	0.26 - 3.56	0.956

176

177 *Alcohol use in the association between trajectories of depressive symptoms on the FH*
178 *and negative outcomes during the SH of TB treatment*

For peer review only - http://bmjopen.bmj.com/site/about/guidelines.xhtml

Alcohol use at least once per week at the start of anti-TB treatment had more LTFU during the SH of TB treatment (OR=2.52; IC95%:1.01-6.28) but the significance was lost when adjusting for trajectories of depressive symptoms, sex, age, marital status, education level and job status (Table 3). After stratification, we found that only in the presence of at least weekly use of alcohol, the association between having an increasing trajectory of depressive symptoms during the FH of TB treatment (compared to those with a decreasing trajectory) and LTFU on the SH was still significant (OR=13.3; IC95%:1.32-134.92).

For peer review only

DISCUSSION

Our results suggest that across the FH of full sensitivity TB treatment the trajectories of depression could be characterized as one of decline over time, one of growth over time after the second month of treatment, and one of high initial score with decline over time. Compared to those that had a declining trajectory, those with an increasing trajectory were more likely to present LTFU during the SH of TB treatment (OR=3.47). Drinking alcohol at least weekly was found to be a potential important effect modifier for this association. As all deaths occurred during the FH of treatment no analysis could be done of this outcome.

As important limitations for the study, the instrument used to measure depressive symptoms, a 5 items version of the CES-D which was validated in a similar population, showed to perform with good sensitivity and specific (>90%), however, there has been no local evaluation regarding its performance when multiple measurements are taken over a relatively small period (6 months). Also, no confirmatory assessment of depression was performed, such as an interview by a clinician or a measurement of impairment. The depressive symptoms measured by the short version of the CES-D scale might be related to be diagnosed with a disease with a heavy burden of stigma.

With respect to the population under study, we could only extrapolate this result to individuals receiving treatment for drug sensitive tuberculosis in primary care settings of urban and sub-urban settings, in the presence of a governmental program specifically designed to identify and treat TB in Peru. Also, the sample size of the study might have not been ideal for clearly defining the trajectories of depressive symptoms when small proportions were found. For example, when analyzing the “High initial score with a decline over time” trajectory which was estimated for 11% of individuals, neither the slope nor the quadratic term showed a rejection of the null hypotheses, thus limiting our understanding of the behavior over time of the individuals that started receiving TB treatment with high levels of depressive symptoms. A limitation of statistical power for the logistic regression in this group might also explain why no association was found between this trajectory and LTFU.

For peer review only - http://bmjopen.bmj.com/site/about/guidelines.xhtml

To our knowledge, this is the first study to explore depressive symptom trajectories during TB treatment and its relationship with LTFU during treatment. Other research has studied the behavior of depressive symptoms in other context; for example, *Owora et al.* found four trajectories for depressive symptoms during HIV treatment: low and high chronic symptoms, episodic and slow rise. These trajectories were similar to those found in this study, in which the higher proportion of population corresponded to the "chronic low symptoms" and "decline" trajectories respectively. The main difference was that we found symptoms of at least moderate magnitude at the start of follow up, which decreased and remained low during the 6 month follow up. This difference might be due to our study taking the first measurement during a period very close to de diagnosis of TB while *Owora et al.* study started the follow up at the first visit to the clinic and might not coincide with the diagnosis.

Two trajectories of increasing magnitude of depressive symptoms were also found by *Owora et al.* accounting for nearly 9% of participants, one with a high episodic trajectory in which symptoms increased between the second and third year of follow up and one of sustained growth over the 6 years follow up. In comparison, we found that a higher proportion of the participants on this groups (>20%), and both showed an initial declining of symptoms before they started increasing in magnitude. The difference in scale of follow up (months vs. years), might explain these differences.

Both studies showed that the trajectory of depressive symptoms might affect treatment outcomes. For example, compared to the group of low chronic symptoms, those with an ascending trajectory had a 50% greater chance of a lower CD4 count during follow-up, which is a similar result to that presented in this research showing a higher chance of LTFU [17].

Identified trajectories were also similar to those found by other studies that describe trajectories of depressive symptoms over time in relation to grief after the loss of a spouse. Grief-related studies, such as those by *Maccallum et al.* and *Galatzer-Levy et al.* showed that after the loss of a spouse or child, the trajectories of depressive symptoms remained constant in both high and low scores and patterns of decline and growth [18,19]. We did not find a trajectory of constant low depressive symptoms. The start

For peer review only - http://bmjopen.bmj.com/site/about/guidelines.xhtml

of follow-up in our study initiated very close to the diagnosis and TB treatment onset, which corresponds to higher frequency of depressive symptoms, in which a mourning effect might be present. As the mourning due to the loss of health and the diagnosis of a stigmatized disease such as TB might resolve by antibiotic treatment, exposure, and better information on the prognosis of the disease, we expect that fewer individuals will present a continuous depressive state, compared to mourning due to the death of a loved one [10].

Weekly alcohol use was found to be an important risk factor that doubles the chances of LTFU in an analysis by *Lackey et al*[20]. Alcohol use has been consistently found a risk factor for both tuberculosis disease and low adherence to treatment, even if the heterogeneity in its definition might contribute on some variation on its significance when controlling for illegal drug use [6,21–24]. Although the interaction between alcohol use disorders and depressive syndromes is complex, and the presence of one might increase the chances of the other, our results show that an increasing trajectory of depressive symptoms is only significantly associated with a greater chance of negative outcomes in the strata of at least weekly alcohol consumption.

For peer review only

For peer review only - http://bmjopen.bmj.com/site/about/guidelines.xhtml

CONCLUSSION

At least three trajectories (decline, growth, and high initial score with decline over time) for depressive symptoms might be present in persons during the FH of full sensitivity TB treatment. Those with increasing symptoms might be at a higher risk of LTFU on the final three months of treatment. The role of alcohol use at the start of treatment might modify the effect of this association.

For peer review only

ACKNOWLEDGMENTS

The authors would like to acknowledge the field team of the Tuberculosis Research Unit at the Instituto de Medicina Tropical Alexander von Humbold of the Universidad Peruana Cayetano Heredia.

CONTRIBUTORSHIP STATEMENT

PR and CUG participated during the development and design of the study. PR performed the statistical analysis. All authors reviewed and made relevant intellectual contributions to the manuscript and approved its submission.

COMPETING INTEREST

The authors declare no competing interests that might have influenced this research.

FUNDING

The original study was funded by Peru ICOHRTA Network for AIDS/TB Research Training (NIH Grant 1U2RTW007368-01A1- Fogarty International Center, Lima Peru) (CUG) and partial support from the Belgian Cooperation through a project of Institutional collaboration between the Institute of Tropical Medicine in Antwerp, Belgium and the Instituto de Medicina Tropical Alexander von Humboldt in Lima, Peru (LO). The funders had no role in study design, data collection and analysis, decision to publish, or preparation of the manuscript. PR received the CIENCIACTIVA-CONCYTEC scholarship for the doctorate program in Epidemiological Research Sciences at the Facultad de Salud Pública y Administración, Universidad Peruana Cayetano Heredia.

DATA AVAILABILITY STATEMENT

Data (without individual identification) are available upon reasonably request to the authors.

For peer review only

REFERENCES

1. World Health Organization. Global tuberculosis report 2018 [Internet]. World Health Organization; 2018 [cited 2019 Jun 26]. Available from: https://apps.who.int/iris/bitstream/handle/10665/274453/9789241565646-eng.pdf

2. Smaoui Fourati S, Mzid H, Marouane C, Kammoun S, Messadi-Akrout F. [Multidrug-resistant tuberculosis: epidemiology and risk factors]. Rev Pneumol Clin [Internet]. 2015 Aug [cited 2019 Jun 26];71(4):233–41. Available from: http://www.ncbi.nlm.nih.gov/pubmed/26210878

3. Gómez-Gómez A, Magaña-Aquino M, López-Meza S, Aranda-Álvarez M, Díaz-Ornelas DE, Hernández-Segura MG, et al. Diabetes and Other Risk Factors for Multi-drug Resistant Tuberculosis in a Mexican Population with Pulmonary Tuberculosis: Case Control Study. Arch Med Res [Internet]. 2015 Feb [cited 2019 Jun 26];46(2):142–8. Available from: https://linkinghub.elsevier.com/retrieve/pii/S0188440915000120

4. World Health Organization.Tuberculosis profile. [Internet]. [cited 2022 Jul 28]. Available from: https://worldhealthorg.shinyapps.io/tb_profiles/?_inputs_&lan=%22EN%22&entity_type=%22group%22&group_code=%22global%22

5. Lackey B, Seas C, van der Stuyft P, Otero L. Patient characteristics associated with tuberculosis treatment default: A cohort study in a high-incidence area of Lima, Peru. PLoS ONE. 2015 Jun 5;10(6).

6. Culqui DR, Munayco E. C v., Grijalva CG, Cayla JA, Horna-Campos O, Alva Ch. K, et al. Factores asociados al abandono de tratamiento antituberculoso convencional en Perú. Archivos de Bronconeumología [Internet]. 2012 May [cited 2019 Jun 26];48(5):150–5. Available from: https://linkinghub.elsevier.com/retrieve/pii/S0300289612000075

7. Muture BN, Keraka MN, Kimuu PK, Kabiru EW, Ombeka VO, Oguya F. Factors associated with default from treatment among tuberculosis patients in Nairobi province, Kenya: a case control study. BMC Public Health [Internet]. 2011 Sep 9 [cited 2019 Jun 26];11(1):696. Available from: http://bmcpublichealth.biomedcentral.com/articles/10.1186/1471-2458-11-696

8. Caylà JA, Rodrigo T, Ruiz-Manzano J, Caminero JA, Vidal R, García JM, et al. Tuberculosis treatment adherence and fatality in Spain. Respir Res [Internet]. 2009 Dec 1 [cited 2019 Jun 26];10(1):121. Available from: http://respiratory-research.biomedcentral.com/articles/10.1186/1465-9921-10-121

9. Duraisamy K, Mrithyunjayan S, Ghosh S, Nair SA, Balakrishnan S, Subramoniapillai J, et al. Does Alcohol Consumption during Multidrug-resistant Tuberculosis Treatment Affect Outcome?. A Population-based Study in Kerala,

For peer review only

For peer review only - http://bmjopen.bmj.com/site/about/guidelines.xhtml

India. Ann Am Thorac Soc [Internet]. 2014 Jun [cited 2019 Jun 26];11(5):712–8. Available from: http://www.ncbi.nlm.nih.gov/pubmed/24735096

10. Ugarte-Gil C, Ruiz P, Zamudio C, Canaza L, Otero L, Kruger H, et al. Association of Major Depressive Episode with Negative Outcomes of Tuberculosis Treatment. Pai M, editor. PLoS ONE [Internet]. 2013 Jul 29 [cited 2019 Jun 26];8(7):e69514. Available from: https://dx.plos.org/10.1371/journal.pone.0069514

11. Vega P, Sweetland A, Acha J, Castillo H, Guerra D, Smith Fawzi MC, et al. Psychiatric issues in the management of patients with multidrug-resistant tuberculosis. Int J Tuberc Lung Dis [Internet]. 2004 Jun [cited 2019 Jun 26];8(6):749–59. Available from: http://www.ncbi.nlm.nih.gov/pubmed/15182146

12. Ruiz-Grosso P, Cachay R, de La Flor A, Schwalb A, Ugarte-Gil C. Association between tuberculosis and depression on negative outcomes of tuberculosis treatment: A systematic review and meta-analysis. PLoS ONE. 2020;15(1).

13. Sánchez A. Migraciones Internas en el Perú. Vol. 1. 2015. 15 p.

14. Dirección General de Epidemiología, Ministerio de Salud del Perú. Análisis de la situación epidemiológica de la tuberculosis en el Perú, 2015 [Internet]. Lima; 2016 [cited 2022 Apr 19]. Available from: http://bvs.minsa.gob.pe/local/MINSA/3446.pdf

15. Ruiz-Grosso, Paulo; Loret de Mola, Christian; Arevalo, Martín; Chavez, Kristhy; Huapaya J. Validez y Consistencia Interna de la Versión de 5 ítems de la Escala para Depresión del Centro de Estudios Epidemiológicos (CESD) y la Escala Auto-administrada de Depresón de Zung (EDZ). In: Jornadas Científicas Universidad Peruana Cayetano Heredia. 2009.

16. Jung T, psychology KWS and personality, 2008 undefined. An introduction to latent class growth analysis and growth mixture modeling. Wiley Online LibraryPaperpile [Internet]. 2007 Jan [cited 2022 Apr 19];2(1):302–17. Available from: https://onlinelibrary.wiley.com/doi/abs/10.1111/j.1751-9004.2007.00054.x

17. Owora AH. Major depression disorder trajectories and HIV disease progression: results from a 6-year outpatient clinic cohort. Medicine [Internet]. 2018 Mar [cited 2019 Jun 26];97(12):e0252. Available from: http://Insights.ovid.com/crossref?an=00005792-201803230-00050

18. Maccallum F, Galatzer-Levy IR, Bonanno GA. Trajectories of depression following spousal and child bereavement: A comparison of the heterogeneity in outcomes. Journal of Psychiatric Research [Internet]. 2015 Oct 1 [cited 2020 Jul 27];69:72–9. Available from: https://pubmed.ncbi.nlm.nih.gov/26343597/

19. Galatzer-Levy IR, Bonanno GA. Beyond normality in the study of bereavement: Heterogeneity in depression outcomes following loss in older adults. Social Science and Medicine [Internet]. 2012 Jun [cited 2020 Jul 27];74(12):1987–94. Available from: https://pubmed.ncbi.nlm.nih.gov/22472274/

For peer review only - http://bmjopen.bmj.com/site/about/guidelines.xhtml

20. Lackey B, Seas C, van der Stuyft P, Otero L. Patient Characteristics Associated with Tuberculosis Treatment Default: A Cohort Study in a High-Incidence Area of Lima, Peru. PLOS ONE [Internet]. 2015 Jun 5 [cited 2022 Apr 30];10(6):e0128541. Available from: https://journals.plos.org/plosone/article?id=10.1371/journal.pone.0128541

21. Tola HH, Tol A, Shojaeizadeh D, Garmaroudi G. Tuberculosis Treatment Non-Adherence and Lost to Follow Up among TB Patients with or without HIV in Developing Countries: A Systematic Review. Iranian Journal of Public Health [Internet]. 2015 [cited 2022 Apr 30];44(1):1. Available from: /pmc/articles/PMC4449995/

22. Muture BN, Keraka MN, Kimuu PK, Kabiru EW, Ombeka VO, Oguya F. Factors associated with default from treatment among tuberculosis patients in Nairobi province, Kenya: a case control study. BMC Public Health [Internet]. 2011 [cited 2022 Apr 30];11. Available from: https://pubmed.ncbi.nlm.nih.gov/21906291/

23. Kliiman K, disease AA journal of tuberculosis and lung, 2010 undefined. Predictors and mortality associated with treatment default in pulmonary tuberculosis. ingentaconnect.comPaperpile [Internet]. [cited 2022 Apr 30]; Available from: https://www.ingentaconnect.com/content/iuatld/ijtld/2010/00000014/00000004/art00014

24. Franke MF, Appleton SC, Bayona J, Arteaga F, Palacios E, Llaro K, et al. Risk factors and mortality associated with default from multidrug-resistant tuberculosis treatment. Clin Infect Dis [Internet]. 2008 Jun 15 [cited 2022 Apr 30];46(12):1844–51. Available from: https://pubmed.ncbi.nlm.nih.gov/18462099/

For peer review only

For peer review only - http://bmjopen.bmj.com/site/about/guidelines.xhtml

Figure 1. Trajectories of depressive symptoms on the first half of TB treatment, 3 trajectories model

Figure 2. Trajectories of depressive symptoms on the first half of TB treatment, 4 trajectories model

For peer review only

For peer review only - http://bmjopen.bmj.com/site/about/guidelines.xhtml

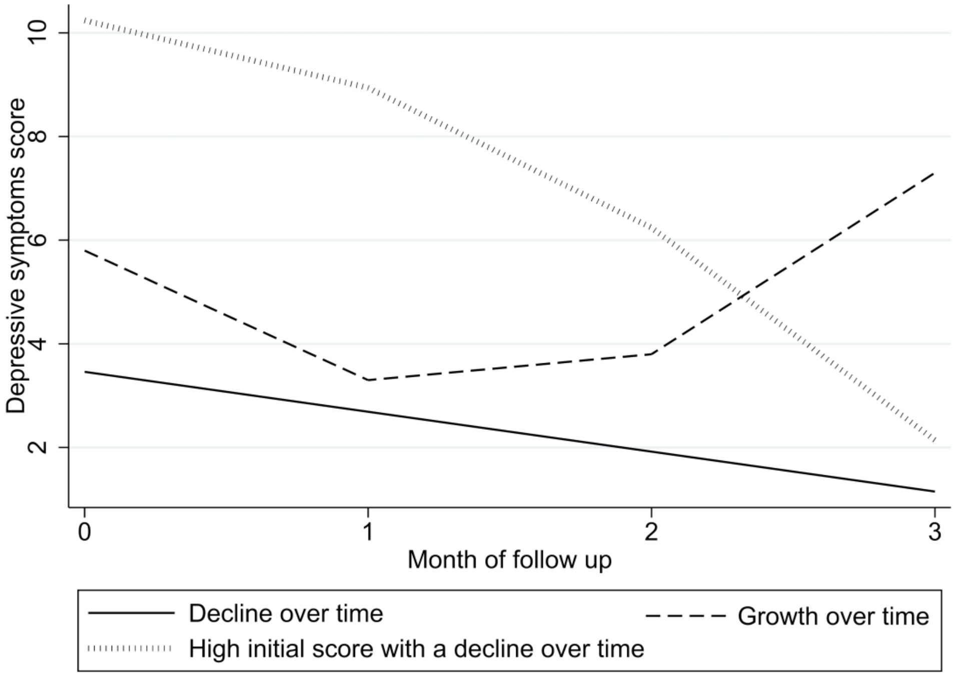

Figure 1

350x254mm (300 x 300 DPI)

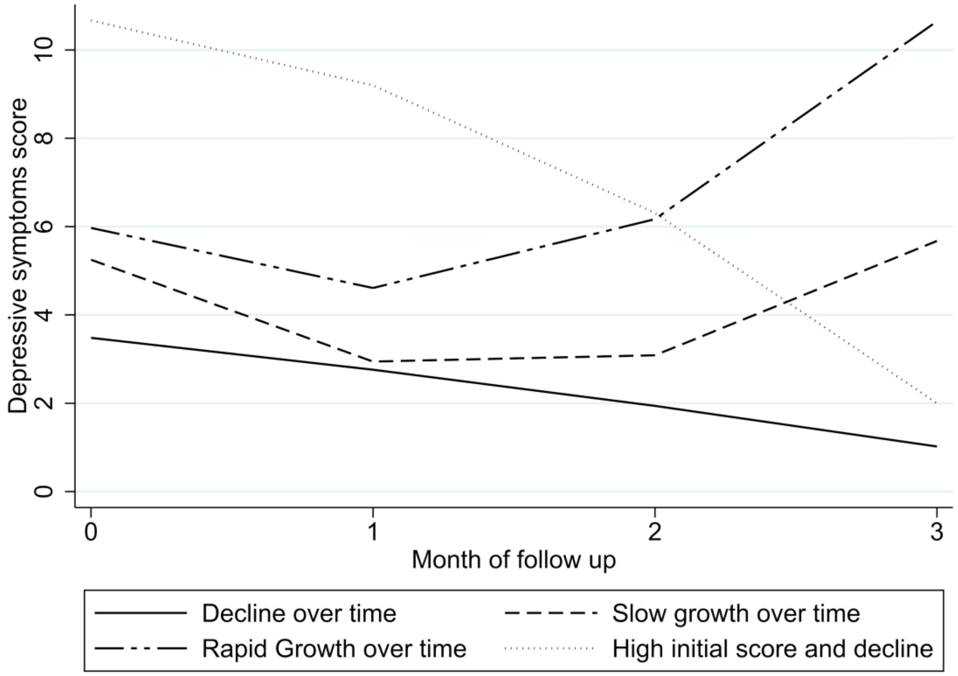

Figure 2.

350x254mm (300 x 300 DPI)

Supplemental Material 1: Goodness of fit of the trajectories of depressive symptoms during the first half of fully sensitive TB treatment.

	**2 trajectories**		**3 trajectories**		**4 trajectories**	
	**Linear**	**Quadratic**	**Linear**	**Quadratic**	**Linear**	**Quadratic**
**AIC**	5448.7	5440	5400.3	5362.7	5351.9	5304.6
**BIC**	5480.9	5482.9	5443.2	5420	5405.6	5376.2
**SSA-BIC**	5452.3	5444.9	5443.2	5369.2	5358.1	5312.7
**Entropy**	0.803	0.828	0.864	0.858	0.909	0.913
**VLR Lrtest***	0.024	0.188	0.003	0.035	0.001	0.001
**BLRT****	<0.001	<0.001	<0.001	<0.001	<0.001	<0.001

*** Vuong–Lo–Mendell–Rubin likelihood ratio test for the null hypothesis that the model with the current number of trajectories is equal that the one with one less trajectory.**

**** Parametric Likelihood Ratio Test for the null hypothesis that the model with the current number of trajectories is equal that the one with one less trajectory using bootstrap.**

For peer review only

STROBE Statement—Checklist of items that should be included in reports of ***cohort studies***

	Item No	Recommendation	Page No
**Title and abstract**	1	(*a*) Indicate the study's design with a commonly used term in the title or the abstract<br>(*b*) Provide in the abstract an informative and balanced summary of what was done and what was found	1 - 3
**Introduction**			
Background/rationale	2	Explain the scientific background and rationale for the investigation being reported	5 - 6
Objectives	3	State specific objectives, including any prespecified hypotheses	5 - 6
**Methods**			
Study design	4	Present key elements of study design early in the paper	7 (line 105)
Setting	5	Describe the setting, locations, and relevant dates, including periods of recruitment, exposure, follow-up, and data collection	7 (lines 112-127)
Participants	6	(*a*) Give the eligibility criteria, and the sources and methods of selection of participants. Describe methods of follow-up<br>(*b*) For matched studies, give matching criteria and number of exposed and unexposed	7 (lines 116-118)
Variables	7	Clearly define all outcomes, exposures, predictors, potential confounders, and effect modifiers. Give diagnostic criteria, if applicable	7-8 (134-148)
Data sources/ measurement	8*	For each variable of interest, give sources of data and details of methods of assessment (measurement). Describe comparability of assessment methods if there is more than one group	7 (lines 119 – 127)
Bias	9	Describe any efforts to address potential sources of bias	8 (139-143)
Study size	10	Explain how the study size was arrived at	As secondary analysis sample sized was fixed.
Quantitative variables	11	Explain how quantitative variables were handled in the analyses. If applicable, describe which groupings were chosen and why	7-8 (128-143)
Statistical methods	12	(*a*) Describe all statistical methods, including those used to control for confounding<br>(*b*) Describe any methods used to examine subgroups and interactions<br>(*c*) Explain how missing data were addressed<br>(*d*) If applicable, explain how loss to follow-up was addressed<br>(*e*) Describe any sensitivity analyses	7-8 (128-143)
**Results**			
Participants	13*	(a) Report numbers of individuals at each stage of study—eg numbers potentially eligible, examined for eligibility, confirmed eligible, included in the study, completing follow-up, and analysed<br>(b) Give reasons for non-participation at each stage<br>(c) Consider use of a flow diagram	9 (lines 150-152)
Descriptive data	14*	(a) Give characteristics of study participants (eg demographic, clinical, social) and information on exposures and potential confounders	9 (152-155)

For peer review only

		(b) Indicate number of participants with missing data for each variable of interest<br><br>(c) Summarise follow-up time (eg, average and total amount)	AND<br>7<br>107-108
Outcome data	15*	Report numbers of outcome events or summary measures over time	9<br>(157 –<br>173)

For peer review only

Main results	16	(*a*) Give unadjusted estimates and, if applicable, confounder-adjusted estimates and their precision (eg, 95% confidence interval). Make clear which confounders were adjusted for and why they were included<br>(*b*) Report category boundaries when continuous variables were categorized<br>(*c*) If relevant, consider translating estimates of relative risk into absolute risk for a meaningful time period	9 (175-178) and Table 3
Other analyses	17	Report other analyses done—eg analyses of subgroups and interactions, and sensitivity analyses	9 (157-173) and 10 (
**Discussion**			
Key results	18	Summarise key results with reference to study objectives	11 (195-203)
Limitations	19	Discuss limitations of the study, taking into account sources of potential bias or imprecision. Discuss both direction and magnitude of any potential bias	11 (204 – 221)
Interpretation	20	Give a cautious overall interpretation of results considering objectives, limitations, multiplicity of analyses, results from similar studies, and other relevant evidence	14 (269-273
Generalisability	21	Discuss the generalisability (external validity) of the study results	11 (112-115)
**Other information**			
Funding	22	Give the source of funding and the role of the funders for the present study and, if applicable, for the original study on which the present article is based	15(277-281)

*Give information separately for exposed and unexposed groups.

Note: An Explanation and Elaboration article discusses each checklist item and gives methodological background and published examples of transparent reporting. The STROBE checklist is best used in conjunction with this article (freely available on the Web sites of PLoS Medicine at http://www.plosmedicine.org/, Annals of Internal Medicine at http://www.annals.org/, and Epidemiology at http://www.epidem.com/). Information on the STROBE Initiative is available at http://www.strobe-statement.org.

For peer review only

For peer review only - http://bmjopen.bmj.com/site/about/guidelines.xhtml

BMJ Open

Are trajectories of depressive symptoms during the first half of drug-sensitive tuberculosis associated with loss to follow-up? A secondary analysis of longitudinal data.

Journal:	*BMJ Open*
Manuscript ID	bmjopen-2022-068235.R1
Article Type:	Original research
Date Submitted by the Author:	21-Apr-2023
Complete List of Authors:	Ruiz-Grosso, Paulo; Universidad Peruana Cayetano Heredia, Medical School; Estado Peruano Ministerio de Salud, Programa de Control de Tuberculosis. Dirección de Redes Integradas de Salud Lima Norte.<br>Loret de Mola, Christian; Universidade Federal do Rio Grande do Sul, CPIS Grupo de Pesqueisa e Inovação em Saúde<br>Otero, Larissa ; Universidad Peruana Cayetano Heredia, Instituto de Medicina Tropical Alexander von Humboldt<br>Ugarte-Gil, Cesar; Universidad Peruana Cayetano Heredia, Facultad de Medicina Alberto Hurtado; Universidad Peruana Cayetano Heredia, Instituto de Medicina Tropical Alexander von Humboldt
**Primary Subject Heading**:	Epidemiology
Secondary Subject Heading:	Epidemiology, Infectious diseases, Mental health
Keywords:	Tuberculosis < INFECTIOUS DISEASES, Depression & mood disorders < PSYCHIATRY, EPIDEMIOLOGY

SCHOLARONE™
Manuscripts

I, the Submitting Author has the right to grant and does grant on behalf of all authors of the Work (as defined in the below author licence), an exclusive licence and/or a non-exclusive licence for contributions from authors who are: i) UK Crown employees; ii) where BMJ has agreed a CC-BY licence shall apply, and/or iii) in accordance with the terms applicable for US Federal Government officers or employees acting as part of their official duties; on a worldwide, perpetual, irrevocable, royalty-free basis to BMJ Publishing Group Ltd ("BMJ") its licensees and where the relevant Journal is co-owned by BMJ to the co-owners of the Journal, to publish the Work in this journal and any other BMJ products and to exploit all rights, as set out in our licence.

The Submitting Author accepts and understands that any supply made under these terms is made by BMJ to the Submitting Author unless you are acting as an employee on behalf of your employer or a postgraduate student of an affiliated institution which is paying any applicable article publishing charge ("APC") for Open Access articles. Where the Submitting Author wishes to make the Work available on an Open Access basis (and intends to pay the relevant APC), the terms of reuse of such Open Access shall be governed by a Creative Commons licence – details of these licences and which Creative Commons licence will apply to this Work are set out in our licence referred to above.

Other than as permitted in any relevant BMJ Author's Self Archiving Policies, I confirm this Work has not been accepted for publication elsewhere, is not being considered for publication elsewhere and does not duplicate material already published. I confirm all authors consent to publication of this Work and authorise the granting of this licence.

For peer review only

Title:

Are trajectories of depressive symptoms during the first half of drug-sensitive tuberculosis associated with loss to follow-up? A secondary analysis of longitudinal data.

Running title:

Depression trajectories and loss to follow up

Authors

Paulo Ruiz-Grosso 1, 2

Christian Loret de Mola 3

Larissa Otero 1, 2

Cesar Ugarte-Gil 1, 2

1 Instituto de Medicina Tropical Alexander von Humboldt, Universidad Peruana Cayetano Heredia, Lima, Peru

2 Facultad de Medicina, Universidad Peruana Cayetano Heredia, Lima, Peru

3 Grupo de pesquisa e inovação em saúde do programa de pós-graduação em saúde pública da universidade federal do Rio Grande, Brazil

Corresponding author:

Paulo Ruiz-Grosso

Universidad Peruana Cayetano Heredia

Av. Honorio Delgado 430, San Martín de Porres

Email: paulo.ruiz@upch.pe

For peer review only

Text word count:

Total: 5273. Main body (including tables): 3039.

Number of references: 26.

Number of tables: 3.

Number of graphics: 3.

For peer review only - http://bmjopen.bmj.com/site/about/guidelines.xhtml

ABSTRACT

Objective: To identify trajectories of depressive symptoms (DS) in the first half of drug-sensitive pulmonary TB (PTB) and their association with loss to follow-up (LTFU) during its second half.

Design: Secondary analysis of longitudinal data to identify possible trajectories and their association with LTFU.

Setting: First and second-level health centers in San Juan de Lurigancho in Lima, Peru.

Participants: Anonymized data from 265 individuals including monthly measures of depressive symptoms from diagnosis to treatment culmination, diagnosis of multi-drug resistant treatment, LTFU, or death was collected for the study.

Results: Three trajectories of DS, “Declining” “Growth” and “High” were presented in 182/265 (68.7%), 53/265 (20%), and 30/265 (11.3 %) of individuals on the first half of PTB treatment, respectively. Compared to those with a “Declining”, those with a “Growth” trajectory had higher chances of LTFU in the second half of PTB treatment controlling for sociodemographic and at least weekly alcohol use (OR=3.9; 95%CI: 1.09-13.97, p=0.036).

Conclusions: A trajectory of increasing DS in the first half of PTB seems to be related to higher chances of LTFU during its second half.

STRENGTHS AND LIMITATIONS OF THIS STUDY

- We used monthly longitudinal measures of depressive symptoms starting from the time of diagnosis of drug-susceptible TB.
- The trajectories (patterns of change) of depressive symptoms were extracted from the data using Latent Class Growth Analysis.
- The limited sample size and the relatively low incidence of the loss to follow-up do not allow for precise confidence intervals.

For peer review only

- A locally validated, 5-item version of the CES-D scale was used to measure depression, however, there is no information on the reliability of the scale when used multiple times in a relatively short period.

For peer review only

For peer review only - http://bmjopen.bmj.com/site/about/guidelines.xhtml

INTRODUCTION

Tuberculosis (TB) was the first cause of death due to infectious diseases worldwide, with 1.6 million deaths caused by TB in 2021, disproportionately affecting developing countries. After the onset of the COVID-19 pandemic, the detection of new cases of TB dropped, being the first period of increase in mortality due to TB since 2015, representing the second cause of death due to infectious diseases around the globe. The emergence of multidrug-resistant strains (MDR) is an important challenge for TB control, with 3.6% of cases detected around the globe starting treatment for MDR or Extensively drug-resistant TB (XDR). In Peru, 5.9% of cases starting treatment were found to be MDR or XDR TB between 2019 and 2022 and is considered one of the countries at a higher risk[1] .

In Peru, treatment for drug-susceptible and MDR or XDR last for at least 6 and 24 months, respectively. Failure or poor adherence during treatment for drug-susceptible TB increases the risk of developing MDR or XDR strains, making it more expensive, longer, and uncomfortable for the patient [2,3]. Failure and loss to follow-up (LTFU) are considered important causes of non-successful TB treatment, accounting for 10 to 17 % of the cases of drug-susceptible TB and 38% of MDR TB [4,5]. It has been associated with a lower level of education, receiving treatment for MDR TB, and comorbid depressive and substance use disorders [5–9] .

Depressive disorders are more frequent in persons treated for TB than in the general population, with a frequency of symptoms suggestive of a major depressive episode that ranged from 38% at the start of TB treatment and 14% at the end of it, while it was estimated in 7% in population-based studies in Lima[10,11] . In persons treated for MDR-TB, 58% and 33% had symptoms suggesting major depression episode at the start and end of the treatment, respectively [12] . Depressive symptoms corresponding to more severe forms such as major depressive episode was associated with death (OR=2.85; 95%CI: 1.52-5.36) and LTFU (OR=4.26; 95%CI: 2.33-7.79) during treatment for TB, suggesting that they might be a target for interventions to improve TB treatment outcomes [13] .

For peer review only - http://bmjopen.bmj.com/site/about/guidelines.xhtml

In this regard, the identification of clinically and epidemiologically relevant trajectories of depressive symptoms during the first half of treatment might allow the identification of sub-groups in which more robust, specialized expensive interventions might be tested and implemented to improve clinical care and help reach the public health aims. We analyzed data from a cohort of patients during drug-sensitive TB treatment to identify trajectories (patterns of change over time) of depressive symptoms associated with LTFU to identify high-risk groups for these outcomes in which to explore opportunities for interventions.

For peer review only

For peer review only - http://bmjopen.bmj.com/site/about/guidelines.xhtml

METHODS

This is an analysis of data from a sub-sample of a cohort study designed to follow up patients during drug-sensitive pulmonary TB treatment in Lima, Peru. The original study took place at primary or secondary care public health centers and collected information on depressive symptoms monthly from TB diagnosis to the end of TB treatment 6 months after, death, LTFU, or referral to another center. The study aimed to study risk factors for negative outcomes during TB treatment, such as death and default. Persons were invited to participate in the study previous to the start of TB treatment.

Individuals that participated in the study lived in San Juan de Lurigancho, a district on the East side of Lima, which is the most populated district in Peru and are usually migrants or descendants of migrants of a large migration from the Andean regions of Peru [14] . The population ranges from middle class to poverty and has one of the larger burdens of TB in Peru[15] . Individuals younger than 18 years old, those with a former diagnosis of TB, pregnant, illiterate, or with any mental or physical condition that would hinder the informed consent process were excluded from the study.

We included information on individuals that had at least four complete measurements of depressive symptoms (baseline and three-monthly follow-ups) and excluded those who were diagnosed with MDR-TB at any point during follow-up and those who died during the first 3 months of treatment and thus were not at risk during the second half of TB treatment and could not contribute to depressive measures in the first half of TB treatment. As a result, data from 265 individuals were included in the data analysis.

The main outcome variable was LTFU during the second half of TB treatment defined as the interruption of antituberculosis treatment for at least 30 days without medical indication, this information was obtained from the official clinical records as part of the original study. Our main predictor variable was trajectories (patterns of change over time) of depressive symptoms during the first half of TB treatment, identified using the score of 5 items, a locally validated version of the CES-D in Spanish [16] . Participants completed the self-rating scale at the start of TB treatment and monthly afterward. Co-variates including age, sex, marital status, education, employment, and frequency of alcohol use were measured at the start of follow-up.

For peer review only - http://bmjopen.bmj.com/site/about/guidelines.xhtml

We analyzed the data in two steps. First, we used latent class growth analysis (LCGA) to identify significant variations in the pattern of depressive symptoms score across the first 3 months of follow-up [17] . For this analysis we used monthly visits as the scale for the time variable, with the “LISTWISE” option for treating missing data, which was specified with its own designation in the database. The number of random starts were of 500 for the initial stage, while 10 optimizations were used for the final stage were used.

We explored linear and quadratic models for trajectories, as linear models would allow us simpler models without any change in the direction of the trajectory, and quadratic models allow us to explore possible changes in the direction of the trajectory that might allow better understanding. No covariates were used during this part of analysis. The code for the analysis of the selected model can be found in Supplemental Material 1.

Then, we used entropy, root mean squared error of approximation (RMSEA), comparative fit index (CFI), Akaike (AIC), Bayesian (BIC) information criteria, the *Vuong–Lo–Mendell–Rubin* and *Bootstrapped Likelihood Ratio Tests* to select the model which best fitted the data. This step provided us with information on the trajectories (slope, linear and quadratic terms) as well as assigning each participant a trajectory.

In the second step, we used logistic regression to test the hypothesis that, individuals that presented worse trajectories of depressive symptoms during the first half of TB treatment were more likely to LTFU during the second half of TB treatment than those with more favorable trajectories. Multiple logistic regression was used to adjust for potential confounders, including sex, age, marital, education, and employment status. In this analysis, we only included observations with complete information on these co-variates.

At least weekly alcohol use at the start of follow-up (defined as drinking alcoholic beverages at least once per week) was analyzed to explore its role as a potential confounder by adjusting and as an effect modifier using stratification. Mplus 8.0 and Stata 15 were used for data analysis.

For peer review only - http://bmjopen.bmj.com/site/about/guidelines.xhtml

Patient and Public Involvement

Neither patients nor the public was involved in the design, conduct, reporting, or dissemination of this research

For peer review only

For peer review only - http://bmjopen.bmj.com/site/about/guidelines.xhtml

RESULTS

Of 344 participants, 34 were excluded because of MDR-TB, and 45 because of missing data on the depression score during the first half of TB treatment. Of the 265 participants analyzed, 13 (4.9%) were lost to follow-up during the second half of TB treatment. The mean age for participants was 29.6 (sd=12.9, 3 missing values (MV)), 145 (54.7%, 3 MV) were male, and most were single (155, 58%, 4 MV). A total of 112 (42.3%, 4 MV) completed secondary education (high school), while 192 (72.5%, 4MV) were unemployed when starting TB treatment. Details of the sample can be found in Table 1.

Symptoms suggesting major depressive episodes were present in 120 (38.7%) individuals during the baseline evaluation and decreased to 33 (13.5) in the last evaluation, at the end of TB treatment. This information is reported with more detail elsewhere [18]. The overall incidence of major depressive episode symptoms after the initiation of TB treatment was 85 individuals (30.3%).

Table 1. Sample characteristics of persons following drug-susceptible TB treatment in Lima, Peru. (n=265)

	N (%)
**Male**	145
**Sex**	(54.7)
	29.6
**Age at enrollment***	(12.9)
**Marital status**	
	155
Single	(58.5)
	90
Married or cohabitant	(34.0)
Divorced or widowed	20 (7.5)

For peer review only

For peer review only - http://bmjopen.bmj.com/site/about/guidelines.xhtml

**Attained education**	
Incomplete elementary	20 (7.5)
Complete elementary	75 (28.3)
Complete secondary	112 (42.3)
Superior education	58 (21.9)
**Currently employed**	73 (27.55)
**Negative outcomes during the second half of TB treatment**	
Death	0 (0.0%)
Loss to follow up	13 (4.91)
Total	13 (4.91)

* mean

Identification of trajectories for depressive symptoms during the first half of TB treatment

A

Two quadratics models with 3 and 4 identified trajectories presented acceptable goodness of fit parameters (See Table 2). The first identified 3 trajectories: 1) “Decline over time of depressive symptoms” (69%), 2) “Growth over time” (20%), and 3) “High initial score with a decline over time” (11%). The second identified 4 trajectories: 1) “Decline over time of depressive symptoms” (65%), 2) “Slow growth over time” (21%), 3) “Rapid Growth over time” (5%), and 4) “High initial score with decline over time” (9%). Both these models were found significantly different from a solution with one less trajectory in both *Vuong–Lo–Mendell–Rubin* (p=0.035 and 0.001 for the 3 and 4

For peer review only - http://bmjopen.bmj.com/site/about/guidelines.xhtml

trajectories models, respectively) and the *Bootstrapped Likelihood Ratio Test* ($p<0.001$ for both). Details on the goodness of fit estimates and estimated parameters of solutions are found in Supplemental Material 2, while graphics of the trajectories might be found in Figures 1 to 3.

We selected the 3-trajectories solution for the next part of the analysis because the 4 - trajectories model showed two trajectories in which the proportion of participants was less than 10% of the sample, and being both growing trajectories very alike, a 3 trajectories models was more parsimonious.

Table 2. Parameters of estimated trajectories of depressive symptoms during the first half of drug-susceptible TB treatment.

	**1 trajectory**		**2 trajectories**		**3 trajectories**		**4 trajectories**	
	**Lineal**	**Quadratic**	**Lineal**	**Quadratic**	**Lineal**	**Quadratic**	**Lineal**	**Quadratic**
Trajectory 1								
% of class	1.00	1	0.18	0.85	0.08	**0.12**	0.05	0.21
Intercept	4.40	4.71	9.40*	3.87	4.70*	**10.24***	3.92*	5.25*
Slope	-0.655	-1.39	-1.74*	-1.56	1.24*	**-0.6**	2.15*	-3.53*
Quadratic term	NA	0.229	NA	0.37	NA	**-0.7**	NA	1.224*
Trajectory 2								
% of class	NA	NA	0.82	0.15	0.79	**0.2**	0.22	0.09
Intercept	NA	NA	3.33	9.38	3.4	**5.8**	3.65*	10.67*
Slope	NA	NA	-0.44*	-0.42	-0.50	**´-4.00***	0.58*	-0.76
Quadratic term	NA	NA	NA	-0.56	NA	**1.50***	NA	-0.71
Trajectory 3								
% of class	NA	NA	NA	NA	0.13	**0.69**	0.09	0.05
Intercept	NA	NA	NA	NA	10.87*	**3.46***	11.97*	5.97*
Slope	NA	NA	NA	NA	-2.77*	**´-0.77***	-3.34*	-2.82
Quadratic term	NA	NA	NA	NA	NA	**~0**	NA	1.46*
Trajectory 4								
% of class	NA	NA	NA	NA	NA	NA	0.64	0.65
Intercept	NA	NA	NA	NA	NA	NA	3.57*	3.48
Slope	NA	NA	NA	NA	NA	NA	-0.84*	-0.67*

For peer review only - http://bmjopen.bmj.com/site/about/guidelines.xhtml

Quadratic term	NA	NA	NA	NA	NA	NA	NA	-0.05*

* p<0.05

Association between trajectories of depressive symptoms on the first half and negative outcomes during the second half of TB treatment.

Compared to those with “Decline over time of depressive symptoms”, those with “Growth over time” during the first half of TB treatment had 3.74 (95%CI: 1.15-12.14) odds of LTFU during the second half. This remained significant when controlling for age, sex, at least weekly use of alcohol, marriage status, education, and job status (OR=3.90; 95%CI: 1.09-13.97; p=0.036). A small but statistically non-significant association with LTFU was found for individuals with a high initial score with decline over time trajectory when compared to those with “Decline over time of depressive symptoms” (OR=1.28; 95%CI: 0.13-12.54; p=0.829). Details of bivariate analyses can be found in Table 3, including coefficient regression of potential confounders for the consideration of readers.

For peer review

Table 3: Variables associated with negative outcomes of TB during the second half of drug-susceptible TB treatment.

Variables	Simple regression			Multiple regression		
	OR	95%CI	p	OR	95%CI	p
Trajectories of depressive symptoms during the first three months of drug-susceptible TB treatment.						
Decline over time	Ref.	-	-	Ref.	-	-
Growth over time	3.74	1.15 - 12.14	0.028	3.9	1.09 - 13.97	0.036
High initial score with decline over time	1.01	0.18 - 8.71	0.992	1.28	0.13 - 12.54	0.829
**Frequent alcohol use**	2.52	1.01 - 6.3	0.046	3.18	0.87 - 11.62	0.08
**Male sex**	1.35	0.54 - 3.22	0.498	2.21	0.50 - 9.75	0.296

For peer review only - http://bmjopen.bmj.com/site/about/guidelines.xhtml

**Age (years)**	1.01	0.97 - 1.04	0.742	0.98	0.92 - 1.05	0.653
Marital Status						
Single	Ref.	-	-	Ref.	-	-
Married or cohabitant	1.35	0.54 - 3.38	0.518	1.86	0.43 - 8.09	0.405
Divorced or widowed	1.92	0.50 - 7.40	0.343	2.35	0.33 - 16.70	0.392
**Attained education**						
Incomplete elementary	Ref.	-	-	Ref.	-	-
Complete elementary	0.78	0.15 - 4.11	0.766	0.97	0.07 - 13.05	0.983
Complete secondary	0.95	0.19 - 4.63	0.948	1.47	0.11 -18.74	0.768
Superior education	0.92	0.17- 5.07	0.921	2.41	0.15 - 39.50	0.538
**Currently employed**	0.69	0.25 - 1.93	0.482	0.96	0.26 - 3.56	0.956

Alcohol use in the association between trajectories of depressive symptoms in the first half and negative outcomes during the second half of TB treatment

Alcohol use at least once per week at the start of anti-TB treatment had more LTFU during the second half of TB treatment (OR=2.52; 95%CI:1.01-6.28; p=0.046) but the significance was lost when adjusting for trajectories of depressive symptoms, sex, age, marital status, education level and job status (Table 3). After stratification, we found that only in the presence of at least weekly use of alcohol, the association between having an increasing trajectory of depressive symptoms during the first half of TB treatment (compared to those with a decreasing trajectory) and LTFU in the second half was still significant (OR=13.3; 95%CI:1.32-134.92; p=0.028).

For peer review only - http://bmjopen.bmj.com/site/about/guidelines.xhtml

Profiling of missing data

During the first half period of TB treatment 45 (14.5%) did not complete at least one of the required four depression measurements and were excluded from the study. In this group, 21 (50%) individuals were female, with a mean age of 33.1, while the proportions of married, single, and divorced individuals were 40, 37.8, and 13.3%. The total of deaths during follow-up occurred during the first half of TB treatment. Of the 20 events of LTFU, 13/265 (4.9%) happened in the group that was analyzed, and 7/45 (15.5%) in the group of individuals that were excluded from the analysis due to incomplete data on depressive symptoms during the first half of TB treatment.

For peer review only

For peer review only - http://bmjopen.bmj.com/site/about/guidelines.xhtml

DISCUSSION

Our results suggest that increasing depressive symptoms during the first half of drug-susceptible TB treatment is associated with LTFU during its second half when compared to patients whose depressive symptoms decreased during this period. Further, a three trajectories model of depressive symptoms during TB treatment includes decreasing, increasing, and high depressive symptoms with a decrease over time. Also, drinking alcohol at least weekly at the start of TB treatment was found to be a potential effect modifier for this association. As all deaths occurred during the first half of TB treatment, no analysis could be done of this outcome.

These findings might allow both researchers and clinicians to be aware of potential characteristics to identify those that might be at a higher risk of LTFU during the later part of TB treatment. This might also contribute to developing more focused interventions that might target mental disorders such as depressive disorders to contribute to improving TB care.

To our knowledge, this is the first study to explore depressive symptom trajectories during TB treatment and its relationship with LTFU during treatment. Other research has studied the behavior of depressive symptoms in another context; for example, *Owora et al.* found four trajectories for depressive symptoms during HIV treatment: two of these showed low and high symptoms across the period of the study, another one showed episodic symptoms, meaning increases during a period and the other a slow rise of depressive symptoms over time. These trajectories were similar to those found in this study, in which the higher proportion of the population which showed a declining trajectory in our study would correspond to the low symptoms over time .

The main difference was that we found symptoms of at least moderate magnitude at the start of follow-up, which decreased and remained low throughout follow-up. This difference might be due to our study taking the first measurement during a period very close to the diagnosis of TB while *Owora et al.* study started the follow-up at the first visit to the clinic and might not coincide with the diagnosis.

For peer review only - http://bmjopen.bmj.com/site/about/guidelines.xhtml

Two trajectories of increasing magnitude of depressive symptoms were also found by *Owora et al.* accounting for nearly 9% of participants, one with a high episodic trajectory in which symptoms increased between the second and third year of follow-up and one of sustained growth over the 6-year follow-up. In comparison, we found that a higher proportion of the participants in this group (>20%), and showed an initial decline of symptoms before they started increasing in magnitude. The difference in the scale of follow-up (months vs. years), might explain these differences.

Both studies showed that the trajectory of depressive symptoms might affect treatment outcomes. For example, compared to the group of low chronic symptoms, those with an ascending trajectory had a 50% greater chance of a lower CD4 count during follow-up, which is a similar result to that presented in this research showing a higher chance of LTFU [19] .

Identified trajectories were also similar to those found by other studies that describe trajectories of depressive symptoms over time concerning grief after the loss of a spouse. Grief-related studies, such as those by *Maccallum et al.* and *Galatzer-Levy et al.* showed that after the loss of a spouse or child, the trajectories of depressive symptoms remained constant in both high and low scores and patterns of decline and growth [20,21] . We did not find a trajectory of constant low depressive symptoms. The start of follow-up in our study started very close to the diagnosis and TB treatment onset, which corresponds to a higher frequency of depressive symptoms, in which a mourning effect might be present. As the mourning due to the loss of health and the diagnosis of a stigmatized disease such as TB might resolve by antibiotic treatment, exposure, and better information on the prognosis of the disease, we expect that fewer individuals will present a continuous depressive state, compared to mourning due to the death of a loved one [10] .

Weekly alcohol use was found to be an important risk factor that doubles the chances of LTFU in an analysis by *Lackey et al*[22] . Alcohol use has been consistently found a risk factor for both tuberculosis disease and low adherence to treatment, even if the heterogeneity in its definition might contribute to some variation in its significance when controlling for illegal drug use [6,23–26] . Although the interaction between alcohol use

For peer review only - http://bmjopen.bmj.com/site/about/guidelines.xhtml

disorders and depressive syndromes is complex, and the presence of one might increase the chances of the other, our results show that an increasing trajectory of depressive symptoms is only significantly associated with a greater chance of negative outcomes in the strata of at least weekly alcohol consumption.

As important limitations for the study, the instrument used to measure depressive symptoms, a 5 items version of the CES-D which was validated in a similar population, showed to perform with good sensitivity and specificity (>90%), however, there has been no local evaluation regarding its performance when multiple measurements are taken over a relatively small period (6 months). Also, no confirmatory assessment of depression was performed, such as an interview by a clinician or a measurement of impairment. The depressive symptoms measured by the short version of the CES-D scale might be related to being diagnosed with a disease with a heavy burden of stigma. Further, no information was collected on the type of management depressive symptoms that might have been received during TB treatment.

Concerning the population under study, we could only extrapolate this result to individuals receiving treatment for drug-sensitive tuberculosis in primary care settings of urban and sub-urban settings, in the presence of a governmental program specifically designed to identify and treat TB in Peru. Also, the sample size of the study might have not been ideal for clearly defining the trajectories of depressive symptoms when small proportions were found. For example, for the “High initial score with a decline over time” trajectory, which was assigned to 11% of individuals, sample size did not allow to conclusively estimate if there is evidence for change in the trajectory over time, or if persons with this trajectory have a higher chance of LTFU compared to those with a decreasing trajectory. Finally, caution should be taken when interpreting the logistic regression coefficients of covariates, as the study was not designed to properly estimate them.

For peer review only - http://bmjopen.bmj.com/site/about/guidelines.xhtml

CONCLUSION

At least three trajectories (decline, growth, and high initial score with decline over time) for depressive symptoms might be present in persons during the first half of drug-susceptible TB treatment. Those with increasing symptoms might be at a higher risk of LTFU in the final three months of treatment. The role of alcohol use at the start of treatment might modify the effect of this association. Further studies with higher statistical power are needed to improve the precision of our estimates, as well as confirm and expand these results.

For peer review only

For peer review only - http://bmjopen.bmj.com/site/about/guidelines.xhtml

REFERENCES

1. World Health Organization. Global tuberculosis report 2022 [Internet]. World Health Organization; 2018 [cited 2019 Jun 26]. Available from: https://www.who.int/publications/i/item/9789240061729

2. Smaoui Fourati S, Mzid H, Marouane C, Kammoun S, Messadi-Akrout F. [Multidrug-resistant tuberculosis: epidemiology and risk factors]. Rev Pneumol Clin [Internet]. 2015 Aug [cited 2019 Jun 26];71(4):233–41. Available from: http://www.ncbi.nlm.nih.gov/pubmed/26210878

3. Gómez-Gómez A, Magaña-Aquino M, López-Meza S, Aranda-Álvarez M, Díaz-Ornelas DE, Hernández-Segura MG, et al. Diabetes and Other Risk Factors for Multi-drug Resistant Tuberculosis in a Mexican Population with Pulmonary Tuberculosis: Case Control Study. Arch Med Res [Internet]. 2015 Feb [cited 2019 Jun 26];46(2):142–8. Available from: https://linkinghub.elsevier.com/retrieve/pii/S0188440915000120

4. TB profile [Internet]. [cited 2022 Jul 28]. Available from: https://worldhealthorg.shinyapps.io/tb_profiles/?_inputs_&lan=%22EN%22&entity_type=%22group%22&group_code=%22global%22

5. Lackey B, Seas C, van der Stuyft P, Otero L. Patient characteristics associated with tuberculosis treatment default: A cohort study in a high-incidence area of Lima, Peru. PLoS One. 2015 Jun 5;10(6).

6. Culqui DR, Munayco E. C v., Grijalva CG, Cayla JA, Horna-Campos O, Alva Ch. K, et al. Factores asociados al abandono de tratamiento antituberculoso convencional en Perú. Arch Bronconeumol [Internet]. 2012 May [cited 2019 Jun 26];48(5):150–5. Available from: https://linkinghub.elsevier.com/retrieve/pii/S0300289612000075

7. Muture BN, Keraka MN, Kimuu PK, Kabiru EW, Ombeka VO, Oguya F. Factors associated with default from treatment among tuberculosis patients in Nairobi province, Kenya: a case control study. BMC Public Health [Internet]. 2011 Sep 9 [cited 2019 Jun 26];11(1):696. Available from: http://bmcpublichealth.biomedcentral.com/articles/10.1186/1471-2458-11-696

8. Caylà JA, Rodrigo T, Ruiz-Manzano J, Caminero JA, Vidal R, García JM, et al. Tuberculosis treatment adherence and fatality in Spain. Respir Res [Internet]. 2009 Dec 1 [cited 2019 Jun 26];10(1):121. Available from: http://respiratory-research.biomedcentral.com/articles/10.1186/1465-9921-10-121

9. Duraisamy K, Mrithyunjayan S, Ghosh S, Nair SA, Balakrishnan S, Subramoniapillai J, et al. Does Alcohol Consumption during Multidrug-resistant Tuberculosis Treatment Affect Outcome?. A Population-based Study in Kerala, India. Ann Am Thorac Soc [Internet]. 2014 Jun [cited 2019 Jun 26];11(5):712–8. Available from: http://www.ncbi.nlm.nih.gov/pubmed/24735096

10. Ugarte-Gil C, Ruiz P, Zamudio C, Canaza L, Otero L, Kruger H, et al. Association of Major Depressive Episode with Negative Outcomes of Tuberculosis Treatment. Pai M, editor. PLoS One [Internet]. 2013 Jul 29 [cited 2019 Jun 26];8(7):e69514. Available from: https://dx.plos.org/10.1371/journal.pone.0069514

11. Profesor H AL, Xvii Año V. ANALES DE ANALES DE SALUD MENTAL SALUD MENTAL HONORIO DELGADO.

For peer review only

Vega P, Sweetland A, Acha J, Castillo H, Guerra D, Smith Fawzi MC, et al. Psychiatric issues in the management of patients with multidrug-resistant tuberculosis. Int J Tuberc Lung Dis [Internet]. 2004 Jun [cited 2019 Jun 26];8(6):749–59. Available from: http://www.ncbi.nlm.nih.gov/pubmed/15182146

Ruiz-Grosso P, Cachay R, de La Flor A, Schwalb A, Ugarte-Gil C. Association between tuberculosis and depression on negative outcomes of tuberculosis treatment: A systematic review and meta-analysis. PLoS One. 2020;15(1).

Sánchez A. Migraciones Internas en el Perú. Vol. 1. 2015. 15 p.

Dirección General de Epidemiología, Ministerio de Salud del Perú. Análisis de la situación epidemiológica de la tuberculosis en el Perú, 2015 [Internet]. Lima; 2016 [cited 2022 Apr 19]. Available from: http://bvs.minsa.gob.pe/local/MINSA/3446.pdf

Ruiz-Grosso, Paulo; Loret de Mola, Christian; Arevalo, Martín; Chavez, Kristhy; Huapaya J. Validez y Consistencia Interna de la Versión de 5 ítems de la Escala para Depresión del Centro de Estudios Epidemiológicos (CESD) y la Escala Auto-administrada de Depresón de Zung (EDZ). In: Jornadas Científicas Universidad Peruana Cayetano Heredia. 2009.

Jung T, psychology KWS and personality, 2008 undefined. An introduction to latent class growth analysis and growth mixture modeling. Wiley Online LibraryPaperpile [Internet]. 2007 Jan [cited 2022 Apr 19];2(1):302–17. Available from: https://onlinelibrary.wiley.com/doi/abs/10.1111/j.1751-9004.2007.00054.x

Ugarte-Gil C, Ruiz P, Zamudio C, Canaza L, Otero L, Kruger H, et al. Association of Major Depressive Episode with Negative Outcomes of Tuberculosis Treatment. PLoS One. 2013 Jul 29;8(7).

Owora AH. Major depression disorder trajectories and HIV disease progression: results from a 6-year outpatient clinic cohort. Medicine [Internet]. 2018 Mar [cited 2019 Jun 26];97(12):e0252. Available from: http://Insights.ovid.com/crossref?an=00005792-201803230-00050

Maccallum F, Galatzer-Levy IR, Bonanno GA. Trajectories of depression following spousal and child bereavement: A comparison of the heterogeneity in outcomes. J Psychiatr Res [Internet]. 2015 Oct 1 [cited 2020 Jul 27];69:72–9. Available from: https://pubmed.ncbi.nlm.nih.gov/26343597/

Galatzer-Levy IR, Bonanno GA. Beyond normality in the study of bereavement: Heterogeneity in depression outcomes following loss in older adults. Soc Sci Med [Internet]. 2012 Jun [cited 2020 Jul 27];74(12):1987–94. Available from: https://pubmed.ncbi.nlm.nih.gov/22472274/

Lackey B, Seas C, van der Stuyft P, Otero L. Patient Characteristics Associated with Tuberculosis Treatment Default: A Cohort Study in a High-Incidence Area of Lima, Peru. PLoS One [Internet]. 2015 Jun 5 [cited 2022 Apr 30];10(6):e0128541. Available from: https://journals.plos.org/plosone/article?id=10.1371/journal.pone.0128541

Tola HH, Tol A, Shojaeizadeh D, Garmaroudi G. Tuberculosis Treatment Non-Adherence and Lost to Follow Up among TB Patients with or without HIV in Developing Countries: A Systematic Review. Iran J Public Health [Internet]. 2015 [cited 2022 Apr 30];44(1):1. Available from: /pmc/articles/PMC4449995/

Muture BN, Keraka MN, Kimuu PK, Kabiru EW, Ombeka VO, Oguya F. Factors associated with default from treatment among tuberculosis patients in Nairobi province, Kenya: a case control study. BMC

For peer review only - http://bmjopen.bmj.com/site/about/guidelines.xhtml

Public Health [Internet]. 2011 [cited 2022 Apr 30];11. Available from: https://pubmed.ncbi.nlm.nih.gov/21906291/

25. Kliiman K, disease AA journal of tuberculosis and lung, 2010 undefined. Predictors and mortality associated with treatment default in pulmonary tuberculosis. ingentaconnect.comPaperpile [Internet]. [cited 2022 Apr 30]; Available from: https://www.ingentaconnect.com/content/iuatld/ijtld/2010/00000014/00000004/art00014

26. Franke MF, Appleton SC, Bayona J, Arteaga F, Palacios E, Llaro K, et al. Risk factors and mortality associated with default from multidrug-resistant tuberculosis treatment. Clin Infect Dis [Internet]. 2008 Jun 15 [cited 2022 Apr 30];46(12):1844–51. Available from: 1. World Health Organization. Global tuberculosis report 2022 [Internet]. World Health Organization; 2018 [cited 2019 Jun 26]. Available from: https://www.who.int/publications/i/item/9789240061729 https://pubmed.ncbi.nlm.nih.gov/18462099/

For peer review only

CONTRIBUTORSHIP STATEMENT

PR was responsible for conceptualization, analysis, interpretation, writing the original draft, review, and editing. CLM was responsible for analysis, interpretation, and critical review. LO was responsible for data acquisition, interpretation, and critical review. CU was responsible for conceptualization, supervision, and critical review. CU is responsible for the overall content as the guarantor.

COMPETING INTEREST

The authors declare no competing interests that might have influenced this research.

FUNDING

The original study was funded by Peru ICOHRTA Network for AIDS/TB Research Training (NIH Grant 1U2RTW007368-01A1- Fogarty International Center, Lima Peru) (CUG) and partial support from the Belgian Cooperation through a project of Institutional collaboration between the Institute of Tropical Medicine in Antwerp, Belgium and the Instituto de Medicina Tropical Alexander von Humboldt in Lima, Peru (LO). The funders had no role in the study design, data collection, analysis, the decision to publish, or the preparation of the manuscript. PR received the CIENCIACTIVA-CONCYTEC scholarship for the doctorate program in Epidemiological Research Sciences at the Facultad de Salud Pública y Administración, Universidad Peruana Cayetano Heredia.

DATA SHARING STATEMENT

Data (without individual identification) are available upon reasonable request to the authors.

ETHICS STATEMENT

Ethical approval for this study was obtained from the Universidad Peruana Cayetano Heredia IRB, under review number (SIDISI number) 203884. All participants participated in the informed consent process and provided informed consent before enrollment in the study. For this study, the data was received without any information that could identify the participants. Treating physicians were informed of the depression status of each participant.

For peer review only - http://bmjopen.bmj.com/site/about/guidelines.xhtml

ACKNOWLEDGMENTS

The authors would like to acknowledge the field team of the Tuberculosis Research Unit at the Instituto de Medicina Tropical Alexander von Humboldt of the Universidad Peruana Cayetano Heredia.

For peer review only

For peer review only - http://bmjopen.bmj.com/site/about/guidelines.xhtml

Figure 1. Trajectories of depressive symptoms on the first half of TB treatment, 3 trajectories model

Figure 2. Trajectories of depressive symptoms on the first half of TB treatment, 4 trajectories model

Figure3. Estimated vs. observed trajectory parameters

For peer review only

For peer review only - http://bmjopen.bmj.com/site/about/guidelines.xhtml

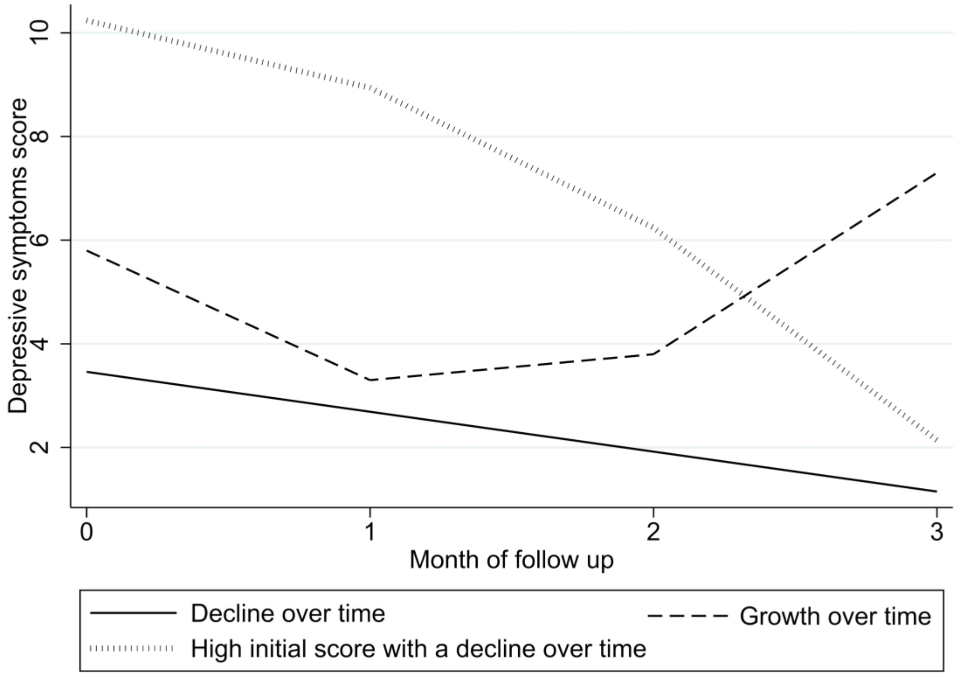

Figure 1

350x254mm (300 x 300 DPI)

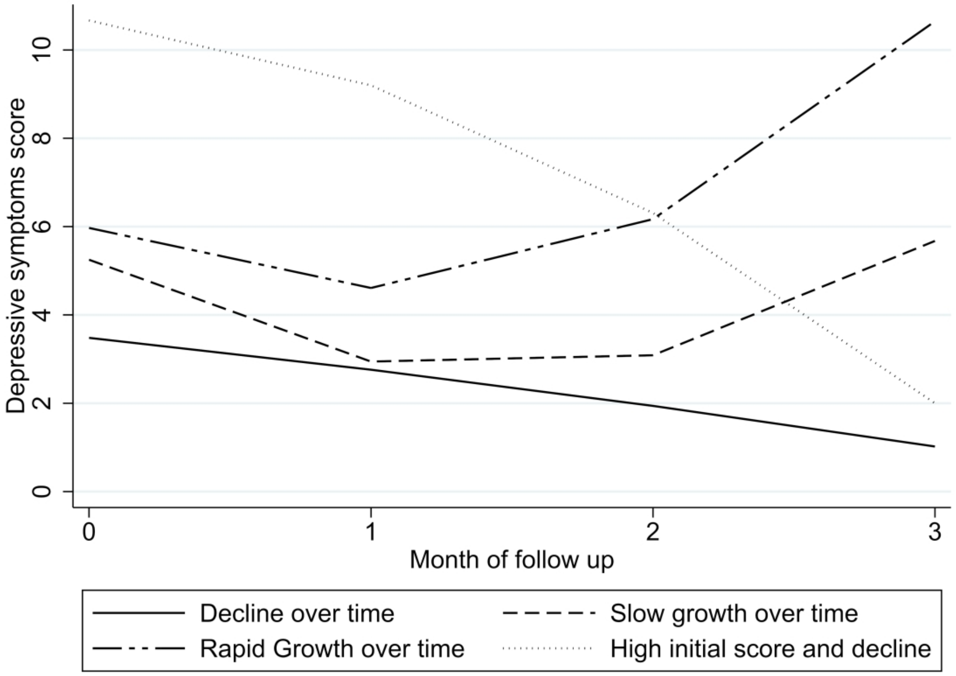

Figure 2.

350x254mm (300 x 300 DPI)

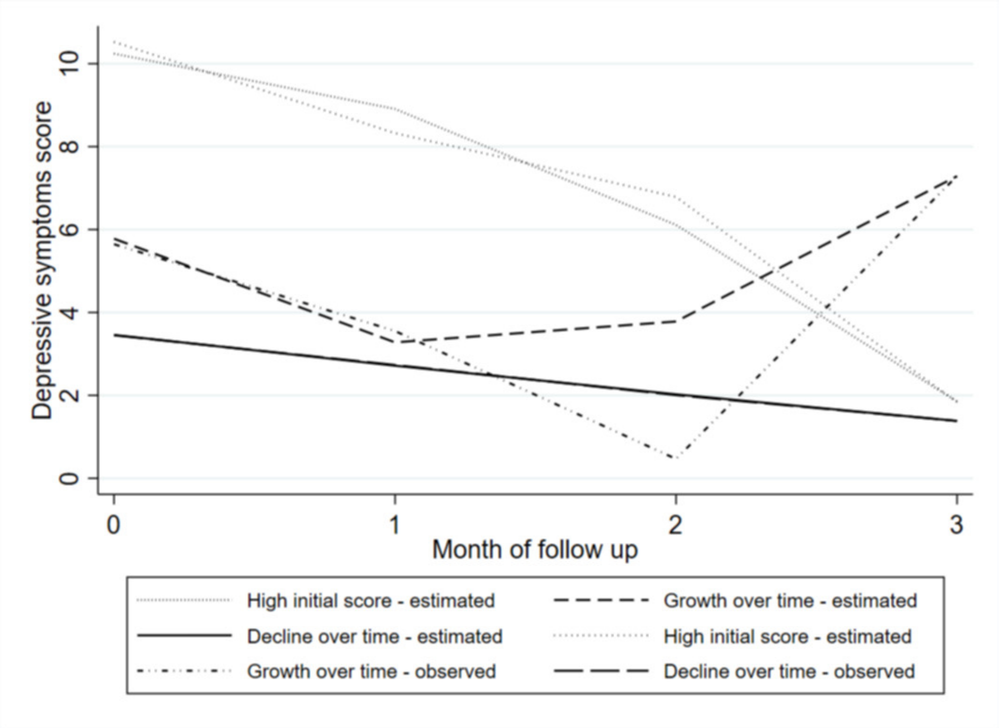

Figure 3

374x272mm (72 x 72 DPI)

Supplemental Material 2: Goodness of fit of the trajectories of depressive symptoms during the first half of fully sensitive TB treatment.

	2 trajectories		3 trajectories		4 trajectories	
	**Linear**	**Quadratic**	**Linear**	**Quadratic**	**Linear**	**Quadratic**
**AIC**	5448.7	5440	5400.3	5362.7	5351.9	5304.6
**BIC**	5480.9	5482.9	5443.2	5420	5405.6	5376.2
**SSA-BIC**	5452.3	5444.9	5443.2	5369.2	5358.1	5312.7
**Entropy**	0.803	0.828	0.864	0.858	0.909	0.913
**VLR Lrtest***	0.024	0.188	0.003	0.035	0.001	0.001
**BLRT****	<0.001	<0.001	<0.001	<0.001	<0.001	<0.001

*** Vuong–Lo–Mendell–Rubin likelihood ratio test for the null hypothesis that the model with the current number of trajectories is equal that the one with one less trajectory.**

**** Parametric Likelihood Ratio Test for the null hypothesis that the model with the current number of trajectories is equal that the one with one less trajectory using bootstrap.**

For peer review only

Supplemental Material 1: Code for performing the trajectory analysis using Mplus v8.0.

```
TITLE:
LCGA cesd0 to cesd3  Quadratic with 3 Classes

DATA: File is
"C:\Users\paulo\Google Drive\Doctorado\Second Try\cesd0-3\cesd3.dta.dat";
    LISTWISE=ON;

VARIABLE:
Names are
     id cesd0 cesd1 cesd2 cesd3 ;
     usevar = cesd0 cesd1 cesd2 cesd3 ;
     IDVARIABLE=id;
     CLASSES=C(3);
  Missing are all (-9999) ;

SAVEDATA: FILE is
"C:\Users\paulo\Google Drive\Doctorado\Second Try\cesd0-3\S2LCGA_0a3_L2.csv";
SAVE=CPROB;

ANALYSIS:
    TYPE=MIXTURE;
    STARTS = 500 10;
    STITERATIONS = 10 ;
    LRTBOOTSTRAP=50;

MODEL: %OVERALL%
I S Q| cesd0@0 cesd1@1 cesd2@2 cesd3@3;
I-S@0;

OUTPUT: SAMPSTAT  STANDARDIZED MOD (5.00) TECH4 TECH7 TECH11 TECH13 TECH14;
PLOT: SERIES = cesd0-cesd3(S); TYPE=PLOT3;
```

For peer review only

For peer review only - http://bmjopen.bmj.com/site/about/guidelines.xhtml

STROBE Statement—Checklist of items that should be included in reports of ***cohort studies***

	Item No	Recommendation	Page No
**Title and abstract**	1	(*a*) Indicate the study's design with a commonly used term in the title or the abstract<br>(*b*) Provide in the abstract an informative and balanced summary of what was done and what was found	1 - 3
**Introduction**			
Background/rationale	2	Explain the scientific background and rationale for the investigation being reported	5 - 6
Objectives	3	State specific objectives, including any prespecified hypotheses	5 - 6
**Methods**			
Study design	4	Present key elements of study design early in the paper	7 (line 105)
Setting	5	Describe the setting, locations, and relevant dates, including periods of recruitment, exposure, follow-up, and data collection	7 (lines 112-127)
Participants	6	(*a*) Give the eligibility criteria, and the sources and methods of selection of participants. Describe methods of follow-up<br>(*b*) For matched studies, give matching criteria and number of exposed and unexposed	7 (lines 116-118)
Variables	7	Clearly define all outcomes, exposures, predictors, potential confounders, and effect modifiers. Give diagnostic criteria, if applicable	7-8 (134-148)
Data sources/ measurement	8*	For each variable of interest, give sources of data and details of methods of assessment (measurement). Describe comparability of assessment methods if there is more than one group	7 (lines 119 – 127)
Bias	9	Describe any efforts to address potential sources of bias	8 (139-143)
Study size	10	Explain how the study size was arrived at	As secondary analysis sample sized was fixed.
Quantitative variables	11	Explain how quantitative variables were handled in the analyses. If applicable, describe which groupings were chosen and why	7-8 (128-143)
Statistical methods	12	(*a*) Describe all statistical methods, including those used to control for confounding<br>(*b*) Describe any methods used to examine subgroups and interactions<br>(*c*) Explain how missing data were addressed<br>(*d*) If applicable, explain how loss to follow-up was addressed<br>(*e*) Describe any sensitivity analyses	7-8 (128-143)
**Results**			
Participants	13*	(a) Report numbers of individuals at each stage of study—eg numbers potentially eligible, examined for eligibility, confirmed eligible, included in the study, completing follow-up, and analysed<br>(b) Give reasons for non-participation at each stage<br>(c) Consider use of a flow diagram	9 (lines 150-152)
Descriptive data	14*	(a) Give characteristics of study participants (eg demographic, clinical, social) and information on exposures and potential confounders	9 (152-155)

For peer review only - http://bmjopen.bmj.com/site/about/guidelines.xhtml

		(b) Indicate number of participants with missing data for each variable of interest<br><br>(c) Summarise follow-up time (eg, average and total amount)	AND<br>7<br>107-108
Outcome data	15*	Report numbers of outcome events or summary measures over time	9<br>(157 –<br>173)

For peer review only

For peer review only - http://bmjopen.bmj.com/site/about/guidelines.xhtml

Main results	16	(*a*) Give unadjusted estimates and, if applicable, confounder-adjusted estimates and their precision (eg, 95% confidence interval). Make clear which confounders were adjusted for and why they were included<br>(*b*) Report category boundaries when continuous variables were categorized<br>(*c*) If relevant, consider translating estimates of relative risk into absolute risk for a meaningful time period	9 (175-178) and Table 3
Other analyses	17	Report other analyses done—eg analyses of subgroups and interactions, and sensitivity analyses	9 (157-173) and 10 (
**Discussion**			
Key results	18	Summarise key results with reference to study objectives	11 (195-203)
Limitations	19	Discuss limitations of the study, taking into account sources of potential bias or imprecision. Discuss both direction and magnitude of any potential bias	11 (204 – 221)
Interpretation	20	Give a cautious overall interpretation of results considering objectives, limitations, multiplicity of analyses, results from similar studies, and other relevant evidence	14 (269-273
Generalisability	21	Discuss the generalisability (external validity) of the study results	11 (112-115)
**Other information**			
Funding	22	Give the source of funding and the role of the funders for the present study and, if applicable, for the original study on which the present article is based	15(277-281)

*Give information separately for exposed and unexposed groups.

Note: An Explanation and Elaboration article discusses each checklist item and gives methodological background and published examples of transparent reporting. The STROBE checklist is best used in conjunction with this article (freely available on the Web sites of PLoS Medicine at http://www.plosmedicine.org/, Annals of Internal Medicine at http://www.annals.org/, and Epidemiology at http://www.epidem.com/). Information on the STROBE Initiative is available at http://www.strobe-statement.org.

For peer review only

BMJ Open

Are trajectories of depressive symptoms during the first half of drug-sensitive pulmonary tuberculosis treatment associated with loss to follow-up? A secondary analysis of longitudinal data

Journal:	*BMJ Open*
Manuscript ID	bmjopen-2022-068235.R2
Article Type:	Original research
Date Submitted by the Author:	11-Jun-2023
Complete List of Authors:	Ruiz-Grosso, Paulo; Universidad Peruana Cayetano Heredia, Medical School; Estado Peruano Ministerio de Salud, Programa de Control de Tuberculosis. Dirección de Redes Integradas de Salud Lima Norte.<br>Loret de Mola, Christian; Universidade Federal do Rio Grande do Sul, CPIS Grupo de Pesqueisa e Inovação em Saúde<br>Otero, Larissa ; Universidad Peruana Cayetano Heredia, Instituto de Medicina Tropical Alexander von Humboldt<br>Ugarte-Gil, Cesar; Universidad Peruana Cayetano Heredia, Facultad de Medicina Alberto Hurtado; Universidad Peruana Cayetano Heredia, Instituto de Medicina Tropical Alexander von Humboldt
**Primary Subject Heading**:	Epidemiology
Secondary Subject Heading:	Epidemiology, Infectious diseases, Mental health
Keywords:	Tuberculosis < INFECTIOUS DISEASES, Depression & mood disorders < PSYCHIATRY, EPIDEMIOLOGY

SCHOLARONE™
Manuscripts

I, the Submitting Author has the right to grant and does grant on behalf of all authors of the Work (as defined in the below author licence), an exclusive licence and/or a non-exclusive licence for contributions from authors who are: i) UK Crown employees; ii) where BMJ has agreed a CC-BY licence shall apply, and/or iii) in accordance with the terms applicable for US Federal Government officers or employees acting as part of their official duties; on a worldwide, perpetual, irrevocable, royalty-free basis to BMJ Publishing Group Ltd ("BMJ") its licensees and where the relevant Journal is co-owned by BMJ to the co-owners of the Journal, to publish the Work in this journal and any other BMJ products and to exploit all rights, as set out in our licence.

The Submitting Author accepts and understands that any supply made under these terms is made by BMJ to the Submitting Author unless you are acting as an employee on behalf of your employer or a postgraduate student of an affiliated institution which is paying any applicable article publishing charge ("APC") for Open Access articles. Where the Submitting Author wishes to make the Work available on an Open Access basis (and intends to pay the relevant APC), the terms of reuse of such Open Access shall be governed by a Creative Commons licence – details of these licences and which Creative Commons licence will apply to this Work are set out in our licence referred to above.

Other than as permitted in any relevant BMJ Author's Self Archiving Policies, I confirm this Work has not been accepted for publication elsewhere, is not being considered for publication elsewhere and does not duplicate material already published. I confirm all authors consent to publication of this Work and authorise the granting of this licence.

For peer review only

Are trajectories of depressive symptoms during the first half of drug-sensitive pulmonary tuberculosis treatment associated with loss to follow-up? A secondary analysis of longitudinal data

Paulo Ruiz-Grosso 1, 2

Christian Loret de Mola 3

Larissa Otero 1, 2

Cesar Ugarte-Gil 1, 2

Affiliations:

1 Instituto de Medicina Tropical Alexander von Humboldt, Universidad Peruana Cayetano Heredia, Lima, Peru

2 Facultad de Medicina, Universidad Peruana Cayetano Heredia, Lima, Peru

3 Grupo de pesquisa e inovação em saúde do programa de pós-graduação em saúde pública da universidade federal do Rio Grande, Brazil

Correspondence to:

Paulo Ruiz-Grosso

Universidad Peruana Cayetano Heredia, Av. Honorio Delgado 430, San Martín de Porres, Lima, Peru

Email: paulo.ruiz@upch.pe

Text word count:

Total: 5275. Main body (including tables): 3956.

Number of references: 26.

Number of tables: 3.

For peer review only - http://bmjopen.bmj.com/site/about/guidelines.xhtml

Number of graphics: 2.

Supplemental Files: 2

For peer review only

For peer review only - http://bmjopen.bmj.com/site/about/guidelines.xhtml

ABSTRACT

Objective: The objective of this study was to identify trajectories of depressive symptoms (DS) during the first half of drug-sensitive pulmonary tuberculosis (PTB) treatment and examine their association with loss to follow-up (LTFU) in the second half.

Design: This study involved a secondary analysis of longitudinal data to identify potential trajectories of DS and their relationship with LTFU.

Setting: The study was conducted in first and second-level health centres located in San Juan de Lurigancho, Lima, Peru.

Participants: Anonymized data from 265 individuals, including monthly measures of depressive symptoms from diagnosis to the completion of treatment, initiation of treatment for multi-drug resistant TB, LTFU, or death, were collected.

Results: Three trajectories were identified: "Declining," "Growth," and "High". These trajectories were observed in 182 (68.7%), 53 (20%), and 30 (11.3%) of the 265 individuals, respectively, during the first half of PTB treatment. Compared to those with a "Declining" trajectory, individuals with a "Growth" trajectory had a higher likelihood of experiencing LTFU during the second half of PTB treatment, after controlling for sociodemographic factors and at least weekly alcohol use (OR=3.9; 95%; CI: 1.09-13.97, p=0.036).

Conclusions: The findings suggest that a trajectory of increasing depressive symptoms during the first half of PTB treatment is associated with a higher risk of LTFU during the second half.

For peer review only - http://bmjopen.bmj.com/site/about/guidelines.xhtml

STRENGTHS AND LIMITATIONS OF THIS STUDY

- We used monthly longitudinal measures of depressive symptoms starting from the time of diagnosis of drug-susceptible pulmonary tuberculosis.
- The trajectories (patterns of change) of depressive symptoms were extracted from the data using Latent Class Growth Analysis.
- The sample size was limited, and incidence of loss to follow-up was relatively low, which may impact the precision of the confidence intervals.
- A locally validated, 5-item version of the CES-D scale was used to measure depression; however, there is no information available regarding the scale´s reliability when used multiple times within a relatively short period.

For peer review only

INTRODUCTION

Tuberculosis (TB) remains the leading cause of death due to infectious diseases worldwide, resulting in 1.6 million deaths in 2021, with a disproportionate impact on developing countries. Following the onset of the COVID-19 pandemic, the detection of new TB cases declined, leading to an increase in TB mortality for the first time since 2015, making it the second leading cause of death from infectious diseases globally. The emergence of multidrug-resistant (MDR) strains poses a significant challenge to TB control, as 3.6% of cases worldwide require treatment for MDR or extensively drug-resistant TB (XDR). In Peru, 5.9% of cases initiating treatment between 2019 and 2022 were found to have MDR or XDR TB, placing the country at the "Higher risk" category by the World Health Organization[1].

In Peru, treatment duration for drug-susceptible TB is at least 6 months, while MDR or XDR treatment extends up to 24 months. Failure or poor adherence during drug-susceptible TB treatment increases the risk of developing MDR or XDR strains, leading to more expensive, lengthier, and uncomfortable treatment for patients[2,3]. Failure and loss to follow-up (LTFU) are significant factors contributing to unsuccessful TB treatment, accounting for 10% to 17% of drug-susceptible TB cases and 38% of MDR TB cases[4,5]. These outcomes have been associated with lower education levels, treatment for MDR TB, and comorbid depressive and substance use disorders[5–9].

Depressive disorders are more prevalent among individuals undergoing TB treatment compared to the general population. In a previously published study among the same cohort, the prevalence of depression at baseline and at the end of TB treatment (after 6 months) was 38% and 14%, respectively; while population-based studies in Lima estimated the prevalence of depression to be close to 7%[10,11]. Among individuals treated for MDR-TB, 58% and 33% exhibited symptoms indicative of a major depressive episode at the start and end of treatment, respectively[12]. Depressive symptoms, including major depressive episodes, have been associated with increased risk of death (OR=2.85; 95%CI: 1.52-5.36) and LTFU (OR=4.26; 95%CI: 2.33-7.79) during TB treatment, suggesting that addressing these symptoms could be crucial for improving TB treatment outcomes[13].

For peer review only - http://bmjopen.bmj.com/site/about/guidelines.xhtml

In this context, identifying clinically and epidemiologically relevant trajectories of depressive symptoms during the first half of pulmonary tuberculosis (PTB) treatment can help identify sub-groups that may benefit from more robust and specialized interventions. These interventions can be tested and implemented to enhance clinical care and achieve public health goals. In this study, we analysed data from a cohort of patients undergoing drug-sensitive TB treatment to identify trajectories (patterns of change over time) of depressive symptoms associated with LTFU. This analysis aims to identify high-risk groups for these outcomes and explore opportunities for interventions.

For peer review only

METHODS

Study design and population

This analysis is based on a sub-sample of a cohort study designed to follow patients undergoing drug-sensitive pulmonary TB treatment in Lima, Peru. The original study was conducted at primary or secondary care public health centres and collected monthly information on depressive symptoms from the time of TB diagnosis until 6 months after the completion of TB treatment, death, LTFU, or referral to another centre. The primary objective of the study was to investigate risk factors associated with negative outcomes during TB treatment, including death and treatment default. Individuals were invited to participate in the study before initiating TB treatment.

The study population consisted of individuals residing in San Juan de Lurigancho, a district located on the East side of Lima. This district is the most populous in Peru and is predominantly composed of migrants or descendants of migrants from the Andean regions of Peru[14]. The population in this area ranges from middle class to impoverished, and the district is one of the most affected with the burden of TB in Peru[15].

Several exclusion criteria were applied in the study. Individuals younger than 18 years old, those with a previous diagnosis of TB, pregnant women, illiterate individuals, and those with any mental or physical condition that could hinder the informed consent process were excluded.

For this analysis, we included information on individuals that had at least four complete measurements of depressive symptoms (baseline and three-monthly follow-ups), and excluded those who were diagnosed with MDR-TB at any point during follow-up and those who died during the first 3 months of treatment and thus were not at risk during the second half of TB treatment and could not contribute to depressive measures in the first half of TB treatment. As a result, data from 265 individuals were included in the data analysis.

Study variables

For peer review only - http://bmjopen.bmj.com/site/about/guidelines.xhtml

The main outcome variable was LTFU during the second half of TB treatment defined as the interruption of antituberculosis treatment for at least 30 days without medical indication, this information was obtained from the official clinical records as part of the original study.

The main predictor variable was trajectories (patterns of change over time) of depressive symptoms during the first half of PTB treatment. These trajectories were identified as part of the data analysis of this study using the score of a 5-item, locally validated version of the CES-D in Spanish[16]. Participants completed this scale at the beginning of TB treatment and then monthly thereafter until treatment completion (6 months), LTFU, or the initiation of treatment for MDR-TB. Each participant contributed between 1 to 7 monthly scores of depressive symptoms.

In addition, several covariates were measured at the start of follow-up, including age, sex, marital status, education, employment status, and frequency of alcohol use.

Statistical analysis

The data analysis was conducted in two steps. First, latent class growth analysis (LCGA) was employed to identify significant variations in the pattern of depressive symptom scores during the first 3 months of follow-up [17]. Monthly visits were used as the time scale variable, and the "LISTWISE" option was utilized to handle missing data, which were designated accordingly in the database. The initial stage of the analysis involved 500 random starts, followed by 10 optimizations in the final stage.

Linear and quadratic models were explored for the trajectories of depressive symptoms. Linear models allowed for simpler models without changes in the direction of the trajectory, while quadratic models allowed for the exploration of possible changes in the trajectory direction for better understanding. No covariates were included in this part of the analysis. The code for the selected model analysis can be found in Supplemental Material 1.

Next, various fit indices, including entropy, root mean squared error of approximation (RMSEA), comparative fit index (CFI), Akaike information criteria (AIC), Bayesian information criteria (BIC), Vuong-Lo-Mendell-Rubin, and Bootstrapped Likelihood Ratio

For peer review only - http://bmjopen.bmj.com/site/about/guidelines.xhtml

Tests, were used to select the model that best fit the data. This step provided information on the trajectories (intercept, linear, and quadratic terms) and assigned each participant to a specific trajectory.

In the second step, logistic regression was employed to test the hypothesis that individuals with worse trajectories of depressive symptoms during the first half of PTB treatment were more likely to experience LTFU during the second half of TB treatment compared to those with more favourable trajectories. Multiple logistic regression was used to adjust for potential confounders, including sex, age, marital status, education, and employment status. Only observations with complete information on these covariates were included in this analysis.

The role of at least weekly alcohol use at the start of follow-up (defined as consuming alcoholic beverages at least once per week) was explored as a potential confounder by adjusting for it and as an effect modifier using stratification.

Patient and public involvement

None.

For peer review only

For peer review only - http://bmjopen.bmj.com/site/about/guidelines.xhtml

RESULTS

Of the initial 344 participants, 34 were excluded due to multidrug-resistant tuberculosis (MDR-TB), and 45 were excluded due to missing data on the depression score during the first half of drug-sensitive pulmonary TB (PTB) treatment. The final analysis included data from 265 participants. Among these participants, 13 (4.9%) were lost to follow-up during the second half of TB treatment.

The mean age of the participants was 29.6 (standard deviation [SD]=12.9), with 3 missing values. Of the analysed participants, 145 (54.7%) were male, with 3 missing values, and the majority were single (155, 58%), with 4 missing values. Among the participants, 112 (42.3%) had completed secondary education (high school), with 4 missing values, while 192 (72.5%) were unemployed at the start of TB treatment, also with 4 missing values. Further details about the sample can be found in Table 1.

During the baseline evaluation, 120 individuals (38.7%) exhibited symptoms suggesting major depressive episodes. This number decreased to 33 (13.5%) in the last evaluation, which occurred at the end of TB treatment. Additional information on this topic is reported in more detail elsewhere[13]. The overall incidence of symptoms suggesting a major depressive episode after the initiation of TB treatment was observed in 85 individuals (30.3%).

Table 1. Sample characteristics of individuals undergoing drug-susceptible TB treatment in Lima, Peru (n=265)

	N (%)
**Male**	145
**Sex**	(54.7)
	29.6
**Age at enrolment***	(12.9)
**Marital status**	

For peer review only

For peer review only - http://bmjopen.bmj.com/site/about/guidelines.xhtml

Single	155 (58.5)
Married or cohabitant	90 (34.0)
Divorced or widowed	20 (7.5)
**Attained education**	
Incomplete elementary	20 (7.5)
Complete elementary	75 (28.3)
Complete secondary	112 (42.3)
Superior education	58 (21.9)
**Currently employed**	73 (27.55)
**Negative outcomes during the second half of TB treatment**	
Death	0 (0.0%)
Loss to follow up	13 (4.91)
Total	13 (4.91)

*Mean.

Identification of trajectories for depressive symptoms during the first half of PTB treatment

Two quadratic models with 3 and 4 identified trajectories demonstrated acceptable goodness-of-fit parameters (refer to Table 2). The first model identified 3 trajectories: 1) "Decline over time of depressive symptoms" (69% of participants), 2) "Growth over time" (20%), and 3) "High initial score with a decline over time" (11%). The second model

For peer review only - http://bmjopen.bmj.com/site/about/guidelines.xhtml

identified 4 trajectories: 1) "Decline over time of depressive symptoms" (65% of participants), 2) "Slow growth over time" (21%), 3) "Rapid growth over time" (5%), and 4) "High initial score with decline over time" (9%). Both models significantly differed from a solution with one less trajectory, as indicated by the *Vuong-Lo-Mendell-Rubin* test (p=0.035 and 0.001 for the 3 and 4 trajectory models, respectively) and the Bootstrapped Likelihood Ratio Test (p<0.001 for both). Additional details on the goodness-of-fit estimates and estimated parameters can be found in Supplemental Material 2, while graphical representations of the trajectories are provided in Figure 1, Figure 2, and Supplemental Figure A.

For peer

For further analysis, we selected the 3-trajectory solution because the 4-trajectory model included two trajectories that accounted for less than 10% of the sample, and the two growth trajectories were quite similar. Thus, the 3-trajectory model was deemed more parsimonious.

Table 2. Parameters of estimated trajectories of depressive symptoms during the first half of drug susceptible PTB treatment

	**1 trajectory**		**2 trajectories**		**3 trajectories**		**4 trajectories**	
	**Linear**	**Quadratic**	**Linear**	**Quadratic**	**Linear**	**Quadratic**	**Linear**	**Quadratic**
Trajectory 1								
% of class	1.00	1	0.18	0.85	0.08	**0.12**	0.05	0.21
Intercept	4.40	4.71	9.40*	3.87	4.70*	**10.24***	3.92*	5.25*
Slope	-0.655	-1.39	-1.74*	-1.56	1.24*	**-0.6**	2.15*	-3.53*
Quadratic term	NA	0.229	NA	0.37	NA	**-0.7**	NA	1.224*
Trajectory 2								
% of class	NA	NA	0.82	0.15	0.79	**0.2**	0.22	0.09
Intercept	NA	NA	3.33	9.38	3.4	**5.8**	3.65*	10.67*
Slope	NA	NA	-0.44*	-0.42	-0.50	**´-4.00***	0.58*	-0.76
Quadratic term	NA	NA	NA	-0.56	NA	**1.50***	NA	-0.71
Trajectory 3								
% of class	NA	NA	NA	NA	0.13	**0.69**	0.09	0.05
Intercept	NA	NA	NA	NA	10.87*	**3.46***	11.97*	5.97*

For peer review only - http://bmjopen.bmj.com/site/about/guidelines.xhtml

Slope	NA	NA	NA	NA	-2.77*	**´-0.77***	-3.34*	-2.82
Quadratic term	NA	NA	NA	NA	NA	**~0**	NA	1.46*
Trajectory 4								
% of class	NA	NA	NA	NA	NA	NA	0.64	0.65
Intercept	NA	NA	NA	NA	NA	NA	3.57*	3.48
Slope	NA	NA	NA	NA	NA	NA	-0.84*	-0.67*
Quadratic term	NA	NA	NA	NA	NA	NA	NA	-0.05*

*p<0.05.

Association between trajectories of depressive symptoms on the first half of PTB and negative outcomes during the second half of PTB treatment

Compared to individuals with a "Decline over time of depressive symptoms" trajectory, those with a "Growth over time" during the first half of PTB treatment had odds of LTFU during the second half that were 3.74 times higher (95%CI: 1.15-12.14). This association remained significant even after controlling for age, sex, at least weekly use of alcohol, marital status, education, and job status (OR=3.90; 95%CI: 1.09-13.97; p=0.036). A small but statistically non-significant association with LTFU was found for individuals with a "High initial score with decline over time" trajectory when compared to those with a "Decline over time of depressive symptoms" (OR=1.28; 95%CI: 0.13-12.54; p=0.829). Details of the bivariate analyses, including coefficient regression of potential confounders, can be found in Table 3 for the consideration of readers.

Table 3. Variables associated with negative outcomes of TB during the second half of drug-susceptible TB treatment

Variables	Simple regression			Multiple regression		
	OR	95%CI	p	OR	95%CI	p
Trajectories of depressive symptoms during the first three months of drug-susceptible TB treatment						
Decline over time	Ref.	-	-	Ref.	-	-

For peer review only - http://bmjopen.bmj.com/site/about/guidelines.xhtml

Growth over time	3.74	1.15 – 12.14	0.028	3.9	1.09 – 13.97	0.036
High initial score with decline over time	1.01	0.18 – 8.71	0.992	1.28	0.13 – 12.54	0.829
**Frequent alcohol use**	2.52	1.01 – 6.3	0.046	3.18	0.87 – 11.62	0.08
**Male sex**	1.35	0.54 – 3.22	0.498	2.21	0.50 – 9.75	0.296
**Age (years)**	1.01	0.97 – 1.04	0.742	0.98	0.92 – 1.05	0.653
Marital Status						
Single	Ref.	-	-	Ref.	-	-
Married or cohabitant	1.35	0.54 – 3.38	0.518	1.86	0.43 – 8.09	0.405
Divorced or widowed	1.92	0.50 – 7.40	0.343	2.35	0.33 – 16.70	0.392
**Attained education**						
Incomplete elementary	Ref.	-	-	Ref.	-	-
Complete elementary	0.78	0.15 – 4.11	0.766	0.97	0.07 – 13.05	0.983
Complete secondary	0.95	0.19 – 4.63	0.948	1.47	0.11 -18.74	0.768
Superior education	0.92	0.17- 5.07	0.921	2.41	0.15 – 39.50	0.538
**Currently employed**	0.69	0.25 – 1.93	0.482	0.96	0.26 – 3.56	0.956

221

222 *Alcohol use in the association between trajectories of depressive symptoms in the first*
223 *half of PTB and negative outcomes during the second half of PTB treatment*

224 Alcohol use at least once per week at the beginning of anti-TB treatment was
225 associated with a higher risk of LTFU during the second half of TB treatment (OR=2.52;
226 95%CI: 1.01-6.28; p=0.046). However, this association lost significance when adjusting

for trajectories of depressive symptoms, sex, age, marital status, education level, and job status (see Table 3).

Upon stratification, we found that the association between having an increasing trajectory of depressive symptoms during the first half of PTB treatment (compared to those with a decreasing trajectory) and LTFU in the second half remained significant only in the presence of at least weekly use of alcohol (OR=13.3; 95%CI: 1.32-134.92; p=0.028)

Profiling of missing data

During the first half of PTB treatment 45 (14.5%) did not complete at least one of the required four depression measurements and were excluded from the study. In this group, 21 (50%) individuals were female, with a mean age of 33.1, while the proportions of married, single, and divorced individuals were 40, 37.8, and 13.3%. The total of deaths during follow-up occurred during the first half of PTB treatment. Of the 20 events of LTFU, 13/265 (4.9%) happened in the group that was analysed, and 7/45 (15.5%) in the group of individuals that were excluded from the analysis due to incomplete data on depressive symptoms during the first half of PTB treatment.

For peer review only - http://bmjopen.bmj.com/site/about/guidelines.xhtml

DISCUSSION

Our findings suggest that an increase in depressive symptoms during the first half of drug susceptible PTB treatment is associated with a higher likelihood of LTFU during the second half, in comparison to patients whose depressive symptoms decreased during this period. We identified three trajectories of depressive symptoms during TB treatment: decreasing, increasing, and high depressive symptoms with a decrease over time.

Furthermore, we found that drinking alcohol at least weekly at the start of TB treatment may modify the association between depressive symptoms trajectories and LTFU. Unfortunately, no analysis of deaths could be conducted as all deaths occurred during the first half of PTB treatment.

These findings have important implications for both researchers and clinicians, as they highlight characteristics that may indicate a higher risk of LTFU in the later stages of TB treatment that might be identified early. This knowledge can contribute to the development of targeted interventions that address mental health disorders, such as depressive and drug use disorders, and ultimately improve TB care.

To the best of our knowledge, this is the first study to investigate the trajectories of depressive symptoms during TB treatment and their association with LTFU during treatment. Previous research has examined the patterns of depressive symptoms in other contexts. For instance, Owora et al. identified four trajectories of depressive symptoms during HIV treatment: low symptoms, high symptoms, episodic symptoms with temporary increases, and a slow increase over time. These trajectories bear similarities to the ones found in our study, with the declining trajectory in our study corresponding to low symptoms over time.

The main difference lies in the presence of at least moderately severe symptoms at the beginning of our follow-up, which subsequently decreased and remained low throughout the study period. This discrepancy may be attributed to our study capturing the first measurement shortly after TB diagnosis, whereas Owora et al.'s study-initiated follow-up at the first clinic visit, which may not coincide with the diagnosis.

For peer review only - http://bmjopen.bmj.com/site/about/guidelines.xhtml

Owora et al. also identified two trajectories of increasing depressive symptoms, accounting for approximately 9% of participants: an episodic trajectory with symptom increases between the second and third year of follow-up, and a sustained growth trajectory over the 6-year follow-up. In comparison, our study revealed a higher proportion (>20%) of participants in this group who initially experienced a decline in symptoms before they started to increase in magnitude. The disparity in follow-up duration (months vs. years) may explain these differences.

Both studies highlight the influence of depressive symptom trajectories on treatment outcomes. For instance, compared to the group with consistently low symptoms, those with an ascending trajectory had a 50% higher likelihood of a lower CD4 count during follow-up, which aligns with our research demonstrating a higher risk of LTFU[18].

The identified trajectories also resemble those observed in studies investigating the trajectory of depressive symptoms following the loss of a spouse. Grief-related studies, such as those by *Maccallum et al.* and *Galatzer-Levy et al.*, demonstrated trajectories characterized by both high and low scores, as well as patterns of decline and growth. However, we did not observe a trajectory of consistently low depressive symptoms. Our study's follow-up commenced shortly after diagnosis and TB treatment initiation, corresponding to a higher frequency of depressive symptoms possibly related to mourning. As the mourning associated with the loss of health and the diagnosis of a stigmatized disease like TB may resolve with antibiotic treatment, exposure, and improved information about the disease prognosis, we anticipate fewer individuals will experience a sustained depressive state compared to mourning following the death of a loved one[10].

Weekly alcohol use was identified as a significant risk factor associated with a twofold increase in the likelihood of LTFU, as reported by *Lackey et al.*[19]. Alcohol use has consistently been recognized as a risk factor for both tuberculosis (TB) disease and poor treatment adherence, although variations in its definition may contribute to differences in significance when controlling for illegal drug use [6,7,20–22]. The interaction between alcohol use disorders and depressive syndromes is complex, and the presence of one may increase the likelihood of the other. However, our findings

For peer review only - http://bmjopen.bmj.com/site/about/guidelines.xhtml

demonstrate that an increasing trajectory of depressive symptoms is only significantly associated with a higher chance of negative outcomes in the subgroup of individuals who engage in at least weekly alcohol consumption.

As important limitations of this study, it should be noted that the instrument used to measure depressive symptoms, a 5-item version of the CES-D scale, was validated in a similar population and demonstrated good sensitivity and specificity (>90%). However, its performance has not been locally evaluated when multiple measurements are taken over a relatively short period of time (6 months). Additionally, no confirmatory assessment of depression, such as a clinician interview or measurement of impairment, was conducted. The measured depressive symptoms using the short version of the CES-D scale may be influenced by the burden of stigma associated with being diagnosed with a disease like tuberculosis. Furthermore, there is no information available regarding whether participants received any therapeutic intervention for depression during TB treatment.

Regarding the study population, it is important to note that the results can only be extrapolated to individuals receiving treatment for drug-sensitive tuberculosis in primary care settings in urban and suburban areas, within the context of a government program specifically designed for identifying and treating TB in Peru. The sample size of the study may not have been ideal for precisely defining trajectories of depressive symptoms when small proportions were observed. For instance, for the "High initial score with a decline over time" trajectory, which was assigned to 11% of individuals, the sample size was not sufficient to conclusively determine if there is evidence of a change in the trajectory over time or if individuals with this trajectory have a higher likelihood of LTFU compared to those with a decreasing trajectory.

Readers should also be aware of the possibility of selection bias influencing the results, as LTFU and possibly the trajectories of depressive disorders may be associated with a higher likelihood of missing data. Finally, caution should be exercised when interpreting adjusted coefficients of the multiple logistic regression model, as even though the coefficient of the main variable may be properly adjusted, the coefficients of covariates

For peer review only - http://bmjopen.bmj.com/site/about/guidelines.xhtml

331 may not be, as discussed by Westreich and Greenland and exemplified by Gretchen et
332 al [23,24].

For peer review only

For peer review only - http://bmjopen.bmj.com/site/about/guidelines.xhtml

CONCLUSION

At least three trajectories (decline, growth, and high initial score with decline over time) of depressive symptoms may be observed in individuals during the first half of drug susceptible PTB treatment. Those with increasing symptoms may be at a heightened risk of LTFU in the final three months of treatment. The presence of alcohol use at the start of treatment may modify this association. Further studies with greater statistical power are necessary to enhance the accuracy of our estimates, as well as to validate and broaden these findings.

For peer review only

For peer review only - http://bmjopen.bmj.com/site/about/guidelines.xhtml

CONTRIBUTORS

PR was responsible for conceptualization, analysis, interpretation, writing the original draft, review, and editing. CLM was responsible for analysis, interpretation, and critical review. LO was responsible for data acquisition, interpretation, and critical review. CU was responsible for conceptualization, supervision, and critical review. CU is responsible for the overall content as the guarantor.

COMPETING INTERESTS

The authors declare no competing interests that might have influenced this research.

FUNDING

The original study was funded by the Peru ICOHRTA Network for AIDS/TB Research Training (NIH Grant 1U2RTW007368-01A1- Fogarty International Center, Lima, Peru) (CUG), with partial support from the Belgian Cooperation through a project of institutional collaboration between the Institute of Tropical Medicine in Antwerp, Belgium, and the Instituto de Medicina Tropical Alexander von Humboldt in Lima, Peru (LO). The funders had no role in the study design, data collection, analysis, the decision to publish, or the preparation of the manuscript. PR received the CIENCIACTIVA (Consejo Nacional de Ciencia, Tecnología e Innovación Tecnológica) scholarship for the Doctorate Program in Epidemiological Research Sciences at the Facultad de Salud Pública y Administración, Universidad Peruana Cayetano Heredia.

DATA AVAILABILITY STATEMENT

Data (without individual identification) are available upon reasonable request to the authors.

ETHICS STATEMENT

Ethical approval for this study was obtained from the Universidad Peruana Cayetano Heredia IRB, under review number (SIDISI number) 203884. All participants participated in the informed consent process and provided informed consent before enrolling in the study. For this study, the data was received without any information that

For peer review only

For peer review only - http://bmjopen.bmj.com/site/about/guidelines.xhtml

could identify the participants. Treating physicians were informed of the depression status of each participant.

ACKNOWLEDGMENTS

The authors would like to acknowledge the field team of the Tuberculosis Research Unit at the Instituto de Medicina Tropical Alexander von Humboldt of the Universidad Peruana Cayetano Heredia.

For peer review only

For peer review only - http://bmjopen.bmj.com/site/about/guidelines.xhtml

REFERENCES

1. World Health Organization. Global tuberculosis report 2022 [Internet]. World Health Organization; 2022 [cited 2023 Jun 6]. Available from: https://reliefweb.int/attachments/249c65c8-2557-49fe-90fa-3988006eb7fe/9789240061729-eng.pdf

2. Smaoui Fourati S, Mzid H, Marouane C, Kammoun S, Messadi-Akrout F. [Multidrug-resistant tuberculosis: epidemiology and risk factors]. Rev Pneumol Clin [Internet]. 2015 Aug [cited 2019 Jun 26];71(4):233–41. Available from: http://www.ncbi.nlm.nih.gov/pubmed/26210878

3. Gómez-Gómez A, Magaña-Aquino M, López-Meza S, Aranda-Álvarez M, Díaz-Ornelas DE, Hernández-Segura MG, et al. Diabetes and Other Risk Factors for Multi-drug Resistant Tuberculosis in a Mexican Population with Pulmonary Tuberculosis: Case Control Study. Arch Med Res [Internet]. 2015 Feb [cited 2019 Jun 26];46(2):142–8. Available from: https://linkinghub.elsevier.com/retrieve/pii/S0188440915000120

4. TB profile [Internet]. [cited 2022 Jul 30]. Available from: https://worldhealthorg.shinyapps.io/tb_profiles/?_inputs_&lan=%22EN%22&entity_type=%22group%22&group_code=%22global%22

5. Lackey B, Seas C, Van Der Stuyft P, Otero L. Patient Characteristics Associated with Tuberculosis Treatment Default: A Cohort Study in a High-Incidence Area of Lima, Peru. PLoS One [Internet]. 2015 Jun 5 [cited 2022 Apr 30];10(6):e0128541. Available from: https://journals.plos.org/plosone/article?id=10.1371/journal.pone.0128541

6. Culqui DR, Munayco E. C V., Grijalva CG, Cayla JA, Horna-Campos O, Alva Ch. K, et al. Factores asociados al abandono de tratamiento antituberculoso convencional en Perú. Arch Bronconeumol [Internet]. 2012 May [cited 2019 Jun 26];48(5):150–5. Available from: https://linkinghub.elsevier.com/retrieve/pii/S0300289612000075

7. Muture BN, Keraka MN, Kimuu PK, Kabiru EW, Ombeka VO, Oguya F. Factors associated with default from treatment among tuberculosis patients in Nairobi province, Kenya: a case control study. BMC Public Health [Internet]. 2011 Sep 9 [cited 2019 Jun 26];11(1):696. Available from: http://bmcpublichealth.biomedcentral.com/articles/10.1186/1471-2458-11-696

8. Caylà JA, Rodrigo T, Ruiz-Manzano J, Caminero JA, Vidal R, García JM, et al. Tuberculosis treatment adherence and fatality in Spain. Respir Res [Internet]. 2009 Dec 1 [cited 2019 Jun 26];10(1):121. Available from: http://respiratory-research.biomedcentral.com/articles/10.1186/1465-9921-10-121

9. Duraisamy K, Mrithyunjayan S, Ghosh S, Nair SA, Balakrishnan S, Subramoniapillai J, et al. Does Alcohol Consumption during Multidrug-resistant Tuberculosis Treatment Affect Outcome?. A Population-based Study in Kerala, India. Ann Am Thorac Soc [Internet]. 2014 Jun [cited 2019 Jun 26];11(5):712–8. Available from: http://www.ncbi.nlm.nih.gov/pubmed/24735096

For peer review only - http://bmjopen.bmj.com/site/about/guidelines.xhtml

10. Ugarte-Gil C, Ruiz P, Zamudio C, Canaza L, Otero L, Kruger H, et al. Association of Major Depressive Episode with Negative Outcomes of Tuberculosis Treatment. PLoS One. 2013 Jul 29;8(7).

11. Saavedra, J. (2013). Estudio Epidemiológico de Salud Mental en Lima Metropolitana y Callao Replicación 2012. In Anales de Salud mental (Vol. 29, No. 1).

12. Vega P, Sweetland A, Acha J, Castillo H, Guerra D, Smith Fawzi MC, et al. Psychiatric issues in the management of patients with multidrug-resistant tuberculosis. Int J Tuberc Lung Dis [Internet]. 2004 Jun [cited 2019 Jun 26];8(6):749–59. Available from: http://www.ncbi.nlm.nih.gov/pubmed/15182146

13. Ruiz-Grosso P, Cachay R, De La Flor A, Schwalb A, Ugarte-Gil C. Association between tuberculosis and depression on negative outcomes of tuberculosis treatment: A systematic review and meta-analysis. PLoS One. 2020;15(1).

14. Sánchez-Aguilar A. Migraciones internas en el Perú. Lima: Organización Internacional para las Migraciones; 2015. p.53.

15. Dirección General de Epidemiología, Ministerio de Salud del Perú. Análisis de la situación epidemiológica de la tuberculosis en el Perú, 2015 [Internet]. Lima; 2016 [cited 2022 Apr 19]. Available from: http://bvs.minsa.gob.pe/local/MINSA/3446.pdf

16. Ruiz-Grosso, Paulo; Loret de Mola, Christian; Arevalo, Martín; Chavez, Kristhy; Huapaya J. Validez y Consistencia Interna de la Versión de 5 ítems de la Escala para Depresión del Centro de Estudios Epidemiológicos (CESD) y la Escala Auto-administrada de Depresón de Zung (EDZ). In: Jornadas Científicas Universidad Peruana Cayetano Heredia. 2009.

17. Jung T, psychology KWS and personality, 2008 undefined. An introduction to latent class growth analysis and growth mixture modeling. Wiley Online LibraryPaperpile [Internet]. 2007 Jan [cited 2022 Apr 19];2(1):302–17. Available from: https://onlinelibrary.wiley.com/doi/abs/10.1111/j.1751-9004.2007.00054.x

18. Owora AH. Major depression disorder trajectories and HIV disease progression: results from a 6-year outpatient clinic cohort. Medicine [Internet]. 2018 Mar [cited 2019 Jun 26];97(12):e0252. Available from: http://Insights.ovid.com/crossref?an=00005792-201803230-00050

19. Lackey B, Seas C, Van Der Stuyft P, Otero L. Patient characteristics associated with tuberculosis treatment default: A cohort study in a high-incidence area of Lima, Peru. PLoS One. 2015 Jun 5;10(6).

20. Tola HH, Tol A, Shojaeizadeh D, Garmaroudi G. Tuberculosis Treatment Non-Adherence and Lost to Follow Up among TB Patients with or without HIV in Developing Countries: A Systematic Review. Iran J Public Health [Internet]. 2015 [cited 2022 Apr 30];44(1):1. Available from: /pmc/articles/PMC4449995/

21. Kliiman K, disease AA journal of tuberculosis and lung, 2010 undefined. Predictors and mortality associated with treatment default in pulmonary tuberculosis. ingentaconnect.comPaperpile

For peer review only

For peer review only - http://bmjopen.bmj.com/site/about/guidelines.xhtml

[Internet]. [cited 2022 Apr 30]; Available from: https://www.ingentaconnect.com/content/iuatld/ijtld/2010/00000014/00000004/art00014

22. Franke MF, Appleton SC, Bayona J, Arteaga F, Palacios E, Llaro K, et al. Risk factors and mortality associated with default from multidrug-resistant tuberculosis treatment. Clin Infect Dis [Internet]. 2008 Jun 15 [cited 2022 Apr 30];46(12):1844–51. Available from: https://pubmed.ncbi.nlm.nih.gov/18462099/

23. Westreich D, Greenland S. The Table 2 Fallacy: Presenting and Interpreting Confounder and Modifier Coefficients. Am J Epidemiol [Internet]. 2013 Feb 2 [cited 2023 May 30];177(4):292. Available from: /pmc/articles/PMC3626058/

24. Bandoli G, Palmsten K, Chambers CD, Jelliffe-Pawlowski LL, Baer RJ, Thompson CA. Revisiting the Table 2 Fallacy: A Motivating Example Examining Preeclampsia and Preterm Birth. Paediatr Perinat Epidemiol [Internet]. 2018 Jul 1 [cited 2023 May 30];32(4):390. Available from: /pmc/articles/PMC6103824/

For peer review only

For peer review only - http://bmjopen.bmj.com/site/about/guidelines.xhtml

FIGURE TITLES (INCLUDING SUPPLEMENTAL FIGURE)

Figure 1. Trajectories of depressive symptoms on the first half of PTB treatment, 3 trajectories model

Figure 2. Trajectories of depressive symptoms on the first half of PTB treatment, 4 trajectories model

Supplemental Figure A. Estimated vs. observed trajectory parameters

For peer review only

For peer review only - http://bmjopen.bmj.com/site/about/guidelines.xhtml

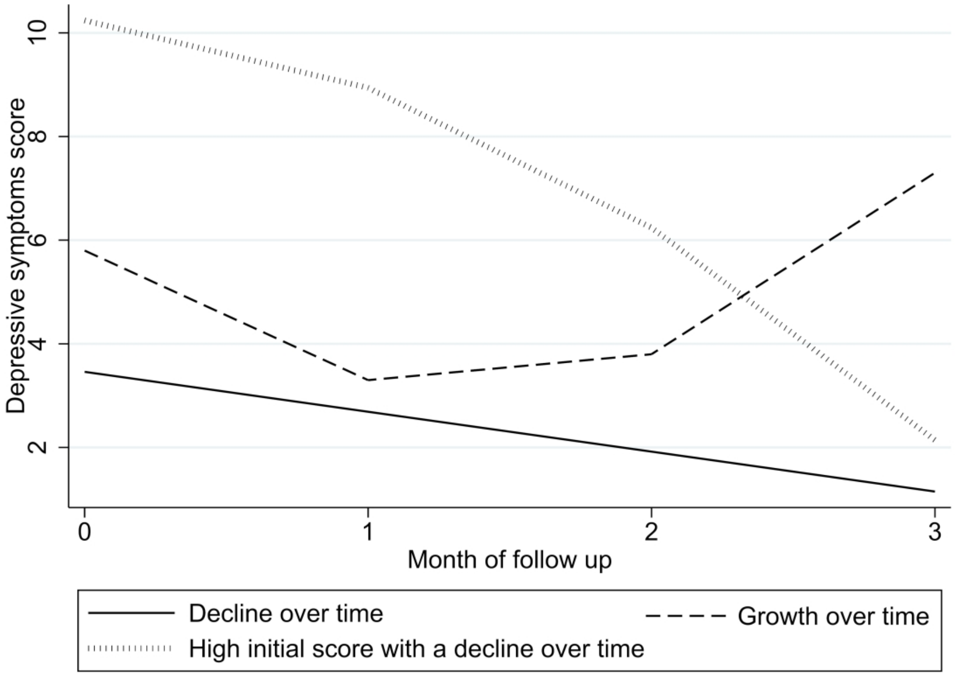

Figure 1

350x254mm (300 x 300 DPI)

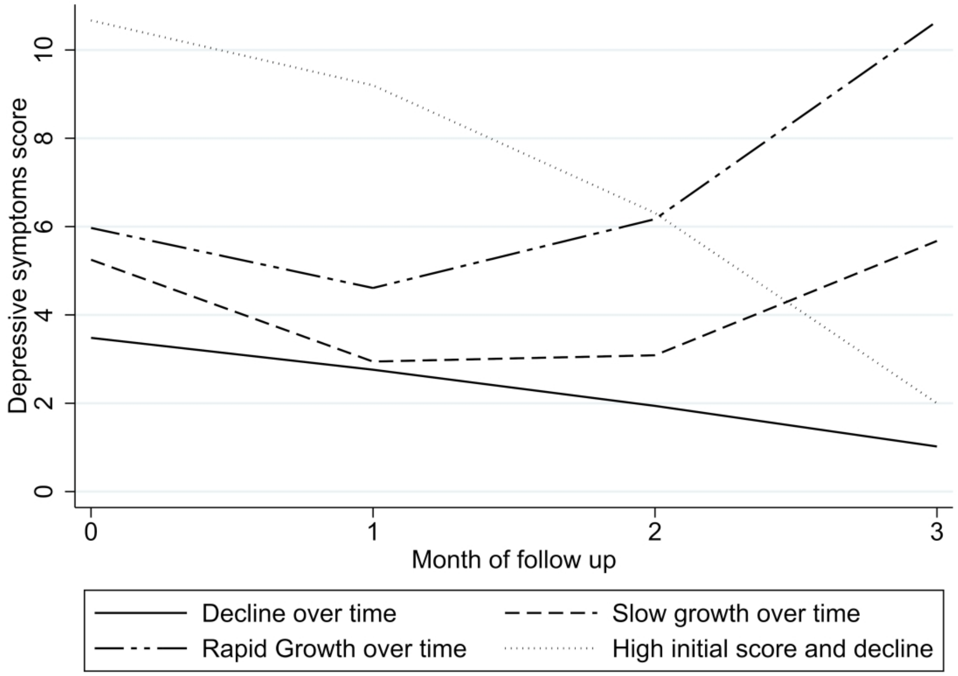

Figure 2.

350x254mm (300 x 300 DPI)

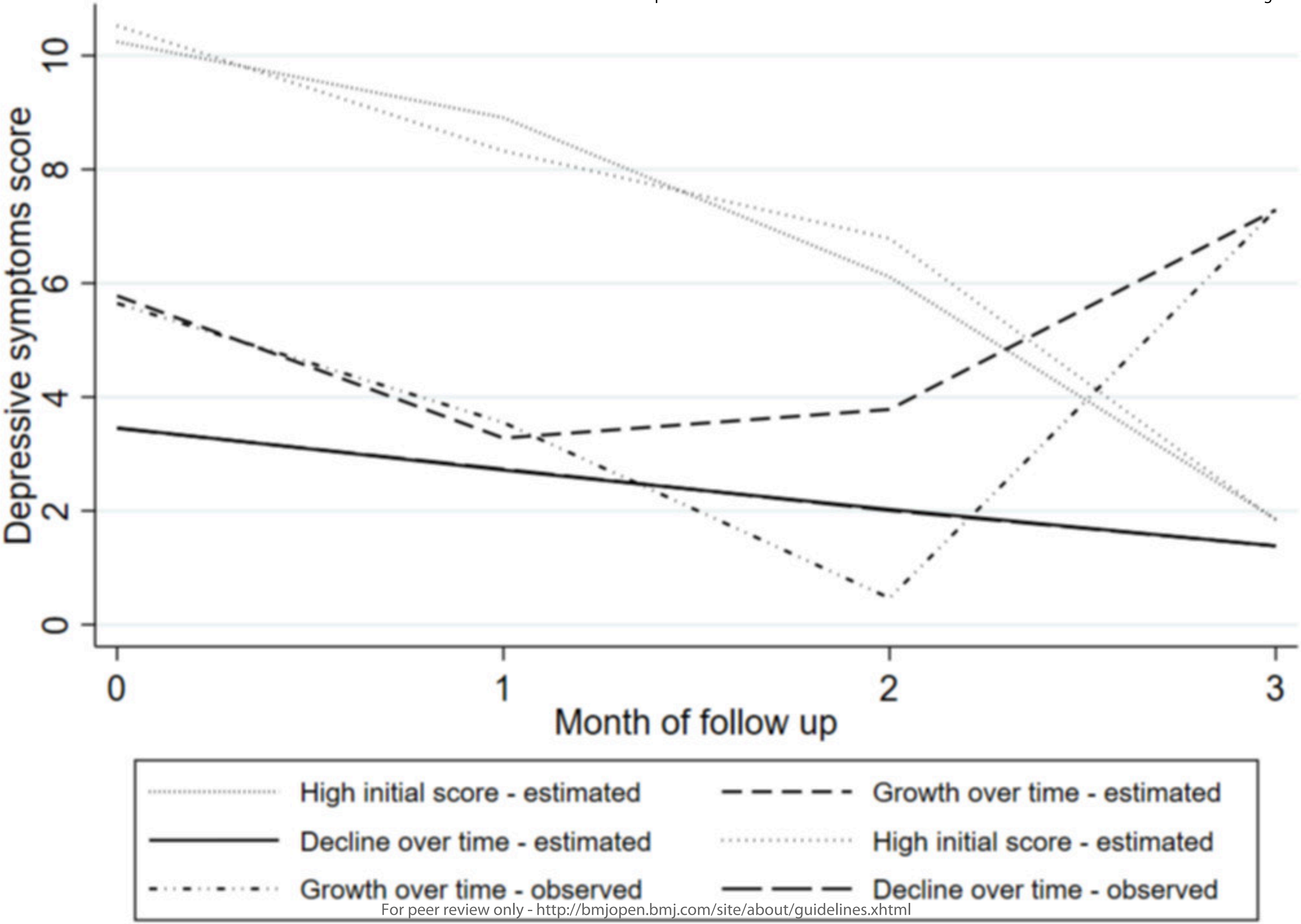
Depressive symptoms score
0
2
4
6
8
10
0
1
2
3
Month of follow up
High initial score - estimated
Growth over time - estimated
Decline over time - estimated
High initial score - estimated
Growth over time - observed
Decline over time - observed

Supplemental Material 1: Code for performing the trajectory analysis using Mplus v8.0.

```
TITLE:
LCGA cesd0 to cesd3  Quadratic with 3 Classes

DATA: File is
"C:\Users\paulo\Google Drive\Doctorado\Second Try\cesd0-3\cesd3.dta.dat";
    LISTWISE=ON;

VARIABLE:
Names are
     id cesd0 cesd1 cesd2 cesd3 ;
     usevar = cesd0 cesd1 cesd2 cesd3 ;
     IDVARIABLE=id;
     CLASSES=C(3);
  Missing are all (-9999) ;

SAVEDATA: FILE is
"C:\Users\paulo\Google Drive\Doctorado\Second Try\cesd0-3\S2LCGA_0a3_L2.csv";
SAVE=CPROB;

ANALYSIS:
    TYPE=MIXTURE;
    STARTS = 500 10;
    STITERATIONS = 10 ;
    LRTBOOTSTRAP=50;

MODEL: %OVERALL%
I S Q| cesd0@0 cesd1@1 cesd2@2 cesd3@3;
I-S@0;

OUTPUT: SAMPSTAT  STANDARDIZED MOD (5.00) TECH4 TECH7 TECH11 TECH13 TECH14;
PLOT: SERIES = cesd0-cesd3(S); TYPE=PLOT3;
```

For peer review only

For peer review only - http://bmjopen.bmj.com/site/about/guidelines.xhtml

Supplemental Material 2: Goodness of fit of the trajectories of depressive symptoms during the first half of fully sensitive TB treatment.

	2 trajectories		3 trajectories		4 trajectories	
	**Linear**	**Quadratic**	**Linear**	**Quadratic**	**Linear**	**Quadratic**
**AIC**	5448.7	5440	5400.3	5362.7	5351.9	5304.6
**BIC**	5480.9	5482.9	5443.2	5420	5405.6	5376.2
**SSA-BIC**	5452.3	5444.9	5443.2	5369.2	5358.1	5312.7
**Entropy**	0.803	0.828	0.864	0.858	0.909	0.913
**VLR Lrtest***	0.024	0.188	0.003	0.035	0.001	0.001
**BLRT****	<0.001	<0.001	<0.001	<0.001	<0.001	<0.001

*** Vuong–Lo–Mendell–Rubin likelihood ratio test for the null hypothesis that the model with the current number of trajectories is equal that the one with one less trajectory.**

**** Parametric Likelihood Ratio Test for the null hypothesis that the model with the current number of trajectories is equal that the one with one less trajectory using bootstrap.**

For peer review only

STROBE Statement—Checklist of items that should be included in reports of ***cohort studies***

	Item No	Recommendation	Page No
**Title and abstract**	1	(*a*) Indicate the study's design with a commonly used term in the title or the abstract<br>(*b*) Provide in the abstract an informative and balanced summary of what was done and what was found	1 - 3
**Introduction**			
Background/rationale	2	Explain the scientific background and rationale for the investigation being reported	5 - 6
Objectives	3	State specific objectives, including any prespecified hypotheses	5 - 6
**Methods**			
Study design	4	Present key elements of study design early in the paper	7 (line 105)
Setting	5	Describe the setting, locations, and relevant dates, including periods of recruitment, exposure, follow-up, and data collection	7 (lines 112-127)
Participants	6	(*a*) Give the eligibility criteria, and the sources and methods of selection of participants. Describe methods of follow-up<br>(*b*) For matched studies, give matching criteria and number of exposed and unexposed	7 (lines 116-118)
Variables	7	Clearly define all outcomes, exposures, predictors, potential confounders, and effect modifiers. Give diagnostic criteria, if applicable	7-8 (134-148)
Data sources/ measurement	8*	For each variable of interest, give sources of data and details of methods of assessment (measurement). Describe comparability of assessment methods if there is more than one group	7 (lines 119 – 127)
Bias	9	Describe any efforts to address potential sources of bias	8 (139-143)
Study size	10	Explain how the study size was arrived at	As secondary analysis sample sized was fixed.
Quantitative variables	11	Explain how quantitative variables were handled in the analyses. If applicable, describe which groupings were chosen and why	7-8 (128-143)
Statistical methods	12	(*a*) Describe all statistical methods, including those used to control for confounding<br>(*b*) Describe any methods used to examine subgroups and interactions<br>(*c*) Explain how missing data were addressed<br>(*d*) If applicable, explain how loss to follow-up was addressed<br>(*e*) Describe any sensitivity analyses	7-8 (128-143)
**Results**			
Participants	13*	(a) Report numbers of individuals at each stage of study—eg numbers potentially eligible, examined for eligibility, confirmed eligible, included in the study, completing follow-up, and analysed<br>(b) Give reasons for non-participation at each stage<br>(c) Consider use of a flow diagram	9 (lines 150-152)
Descriptive data	14*	(a) Give characteristics of study participants (eg demographic, clinical, social) and information on exposures and potential confounders	9 (152-155)

For peer review only - http://bmjopen.bmj.com/site/about/guidelines.xhtml

		(b) Indicate number of participants with missing data for each variable of interest<br><br>(c) Summarise follow-up time (eg, average and total amount)	AND<br>7<br>107-108
Outcome data	15*	Report numbers of outcome events or summary measures over time	9<br>(157 –<br>173)

For peer review only

Main results	16	(*a*) Give unadjusted estimates and, if applicable, confounder-adjusted estimates and their precision (eg, 95% confidence interval). Make clear which confounders were adjusted for and why they were included<br>(*b*) Report category boundaries when continuous variables were categorized<br>(*c*) If relevant, consider translating estimates of relative risk into absolute risk for a meaningful time period	9 (175-178) and Table 3
Other analyses	17	Report other analyses done—eg analyses of subgroups and interactions, and sensitivity analyses	9 (157-173) and 10 (
**Discussion**			
Key results	18	Summarise key results with reference to study objectives	11 (195-203)
Limitations	19	Discuss limitations of the study, taking into account sources of potential bias or imprecision. Discuss both direction and magnitude of any potential bias	11 (204 – 221)
Interpretation	20	Give a cautious overall interpretation of results considering objectives, limitations, multiplicity of analyses, results from similar studies, and other relevant evidence	14 (269-273
Generalisability	21	Discuss the generalisability (external validity) of the study results	11 (112-115)
**Other information**			
Funding	22	Give the source of funding and the role of the funders for the present study and, if applicable, for the original study on which the present article is based	15(277-281)

*Give information separately for exposed and unexposed groups.

Note: An Explanation and Elaboration article discusses each checklist item and gives methodological background and published examples of transparent reporting. The STROBE checklist is best used in conjunction with this article (freely available on the Web sites of PLoS Medicine at http://www.plosmedicine.org/, Annals of Internal Medicine at http://www.annals.org/, and Epidemiology at http://www.epidem.com/). Information on the STROBE Initiative is available at http://www.strobe-statement.org.

For peer review only

For peer review only - http://bmjopen.bmj.com/site/about/guidelines.xhtml
